# Microbiome and metabolome features of the cardiometabolic disease spectrum

**DOI:** 10.1038/s41591-022-01688-4

**Published:** 2022-02-17

**Authors:** Sebastien Fromentin, Sofia K. Forslund, Kanta Chechi, Judith Aron-Wisnewsky, Rima Chakaroun, Trine Nielsen, Valentina Tremaroli, Boyang Ji, Edi Prifti, Antonis Myridakis, Julien Chilloux, Petros Andrikopoulos, Yong Fan, Michael T. Olanipekun, Renato Alves, Solia Adiouch, Noam Bar, Yeela Talmor-Barkan, Eugeni Belda, Robert Caesar, Luis Pedro Coelho, Gwen Falony, Soraya Fellahi, Pilar Galan, Nathalie Galleron, Gerard Helft, Lesley Hoyles, Richard Isnard, Emmanuelle Le Chatelier, Hanna Julienne, Lisa Olsson, Helle Krogh Pedersen, Nicolas Pons, Benoit Quinquis, Christine Rouault, Hugo Roume, Joe-Elie Salem, Thomas S. B. Schmidt, Sara Vieira-Silva, Peishun Li, Maria Zimmermann-Kogadeeva, Christian Lewinter, Nadja B. Søndertoft, Tue H. Hansen, Dominique Gauguier, Jens Peter Gøtze, Lars Køber, Ran Kornowski, Henrik Vestergaard, Torben Hansen, Jean-Daniel Zucker, Serge Hercberg, Ivica Letunic, Fredrik Bäckhed, Jean-Michel Oppert, Jens Nielsen, Jeroen Raes, Peer Bork, Michael Stumvoll, Eran Segal, Karine Clément, Marc-Emmanuel Dumas, S. Dusko Ehrlich, Oluf Pedersen

**Affiliations:** 1 MetaGenoPolis, INRAe, AgroParisTech, Université Paris-Saclay, Paris, France; 2 Structural and Computational Biology, European Molecular Biology Laboratory, Heidelberg, Germany; 3 Experimental and Clinical Research Center, a cooperation of Charité-Universitätsmedizin and the Max-Delbrück Center, Berlin, Germany; 4 Max Delbrück Center for Molecular Medicine (MDC), Berlin, Germany; 5 Charité University Hospital, Berlin, Germany; 6 DZHK (German Centre for Cardiovascular Research), Partner Site Berlin, Berlin, Germany; 7 Section of Biomolecular Medicine, Division of Systems Medicine, Department of Metabolism, Digestion and Reproduction, Faculty of Medicine, Imperial College London, London, UK; 8 School of Public Health, Faculty of Medicine, Imperial College London, Medical School Building, St. Mary’s Hospital, London, UK; 9 Genomic and Environmental Medicine, National Heart and Lung Institute, Faculty of Medicine, Imperial College London, London, UK; 10 Sorbonne Université, INSERM, Nutrition and obesities; systemic approaches (NutriOmics), Paris, France; 11 Assistance Publique Hôpitaux de Paris, Pitié-Salpêtrière Hospital, Nutrition Department, Paris, France; 12 Medical Department III - Endocrinology, Nephrology, Rheumatology, University of Leipzig Medical Center, Leipzig, Germany; 13 Novo Nordisk Foundation Center for Basic Metabolic Research, Faculty of Health and Medical Sciences, University of Copenhagen, Copenhagen, Denmark; 14 The Wallenberg Laboratory, Department of Molecular and Clinical Medicine, Institute of Medicine, Sahlgrenska Academy, University of Gothenburg, Gothenburg, Sweden; 15 Department of Biology and Biological Engineering, Chalmers University of Technology, Gothenburg, Sweden; 16 Unité de modélisation mathématique et informatique des systèmes complexes, UMMISCO, Bondy, France; 17 Department of Computer Science and Applied Mathematics, Weizmann Institute of Science, Rehovot, Israel; 18 Department of Molecular Cell Biology, Weizmann Institute of Science, Rehovot, Israel; 19 Department of Cardiology, Rabin Medical Center, Petah Tikva, Israel; 20 Sackler Faculty of Medicine, Tel-Aviv University, Tel-Aviv, Israel; 21 Institute of Cardiometabolism and Nutrition, Integromics Unit, Paris, France; 22 Integrative Phenomics, Paris, France; 23 Laboratory of Molecular Bacteriology, Department of Microbiology and Immunology, Rega Institute, KU Leuven, Leuven, Belgium; 24 Center for Microbiology, VIB, Leuven, Belgium; 25 Assistance Publique-Hôpitaux de Paris, Hôpitaux Universitaires Henri Mondor, Département de biochimie-pharmacologie-biologie moléculaire-génétique médicale, Créteil, France; 26 Assistance Publique Hôpitaux de Paris, Pitié-Salpêtrière Hospital, Cardiology Department, Paris, France; 27 Nottingham Trent University, Department of Bioscience, School of Science and Technology, Nottingham, UK; 28 AP-HP, Pitié-Salpêtrière Hospital, Department of Pharmacology, UNICO Cardio-oncology Program, CIC-1421; INSERM, Sorbonne Université, Paris, France; 29 Department of Cardiology, Rigshospitalet, University of Copenhagen, Copenhagen, Denmark; 30 Université de Paris, INSERM UMR 1124, Paris, France; 31 Department of Clinical Biochemetry, Rigshopitalet, University of Copenhagen, Copenhagen, Denmark; 32 Department of Medicine, Bornholms Hospital, Rønne, Denmark; 33 Sorbonne Paris Cité Epidemiology and Statistics Research Centre (CRESS), U1153 Inserm, U1125, Inra, Cnam, University of Paris 13, Nutritional Epidemiology Research Team (EREN), Bobigny, France; 34 Biobyte Solutions GmbH, Heidelberg, Germany; 35 European Genomics Institute for Diabetes, UMR1283/8199 INSERM, CNRS, Institut Pasteur de Lille, Lille University Hospital, University of Lille, Lille, France; 36 Department of Clinical and Movement Neurosciences, University College London, London, UK

**Keywords:** Cardiovascular diseases, Metabolic syndrome

## Abstract

Previous microbiome and metabolome analyses exploring non-communicable diseases have paid scant attention to major confounders of study outcomes, such as common, pre-morbid and co-morbid conditions, or polypharmacy. Here, in the context of ischemic heart disease (IHD), we used a study design that recapitulates disease initiation, escalation and response to treatment over time, mirroring a longitudinal study that would otherwise be difficult to perform given the protracted nature of IHD pathogenesis. We recruited 1,241 middle-aged Europeans, including healthy individuals, individuals with dysmetabolic morbidities (obesity and type 2 diabetes) but lacking overt IHD diagnosis and individuals with IHD at three distinct clinical stages—acute coronary syndrome, chronic IHD and IHD with heart failure—and characterized their phenome, gut metagenome and serum and urine metabolome. We found that about 75% of microbiome and metabolome features that distinguish individuals with IHD from healthy individuals after adjustment for effects of medication and lifestyle are present in individuals exhibiting dysmetabolism, suggesting that major alterations of the gut microbiome and metabolome might begin long before clinical onset of IHD. We further categorized microbiome and metabolome signatures related to prodromal dysmetabolism, specific to IHD in general or to each of its three subtypes or related to escalation or de-escalation of IHD. Discriminant analysis based on specific IHD microbiome and metabolome features could better differentiate individuals with IHD from healthy individuals or metabolically matched individuals as compared to the conventional risk markers, pointing to a pathophysiological relevance of these features.

## Abstract (web summary)

By studying individuals along a spectrum of cardiometabolic disease and adjusting for effects of lifestyle and medication, this investigation identifies alterations of the metabolome and microbiome from dysmetabolic conditions, such as obesity and type 2 diabetes, to ischemic heart disease.

## Main

Epidemiological and genetic studies in humans and experimental studies in animals have shown that the pathogenesis of most common chronic non-communicable diseases involves a complex interplay among polygenic susceptibility, aging, sex and a multitude of environmental exposures^1^. Intriguingly, environmental components, such as diet, physical activity and smoking, might exert some of their pathogenic effect via modification of the intestinal microbiome^2^. Therefore, a first logical step in exploration of the intestinal microbiome as a putative chronic disease co-trigger appears to be the conduct of studies integrating epidemiology and various -omics analyses. However, for the reliability of such study outcomes and for the planning of subsequent clinical interventions and mechanistic experiments, disease-specific microbiome and linked metabolome features need to be separated from confounders introduced by pre-morbidities and co-morbidities^3–5^ and by multifactorial treatment^6^. Commonly prescribed drugs, for example, widely influence the gut microbiome and host metabolome^7^ and can confuse for, or even mask, genuine disease signatures^7,8^. Accordingly, a recent report argues for extensive adjustments for confounders that influence the human gut microbiome to avoid spurious associations and to identify genuine disease-specific variance^9^.

The present microbiome and metabolome study is focused on IHD, a leading cause of mortality worldwide^10^. Previous reports comparing microbiome and metabolome markers of IHD cases and controls usually failed to adjust for the massive confounding by polypharmacy^8^ and the effect of metabolic abnormalities occurring during a long prodromal phase before diagnosis of IHD^11–13^. Such common metabolic dysfunctions include overweight and obesity^3,5^, type 2 diabetes (T2D)^4^, hypertension^14^ and dyslipidaemia^15^ (collectively termed ‘dysmetabolism’ in the present study), all of which have been shown to exhibit both shared and disease-specific aberrations in microbiome and metabolome profiles. Individuals with the metabolic syndrome or overt T2D have vastly increased risk of IHD^16^, and asymptomatic T2D is often coincidentally found at IHD diagnosis^17^, highlighting these pre-morbidities to be a clinically relevant baseline for studying overt IHD. Most studies to date have overlooked this aspect by either comparing individuals with IHD with healthy, lean individuals^11^ or not focusing on IHD per se but on various forms of atherosclerotic organ damage^12,13,18^. Thus, segregating IHD-specific changes in gut microbial and metabolomic features from such potential confounders remains an utmost priority.

In the MetaCardis consortium, we designed the present cross-sectional study including healthy individuals, individuals with dysmetabolic morbidities and individuals with IHD at three distinct clinical stages, capturing a wide spectrum of gut microbiome and plasma and urine metabolome signatures for cardiometabolic diseases (CMDs). With our approach for integrative analysis of the -omics data, we adjust for confounding by polypharmacy and the effect of metabolic abnormalities occurring during the prodromal phase before diagnosis of IHD. Furthermore, we categorize microbiome and metabolome pathophysiological signatures related to dysmetabolism or to escalation, de-escalation or stabilization of IHD and its subtypes.

## Results

### Study design, in-depth phenotyping and multi-omics profiling

**Fig. 1:**
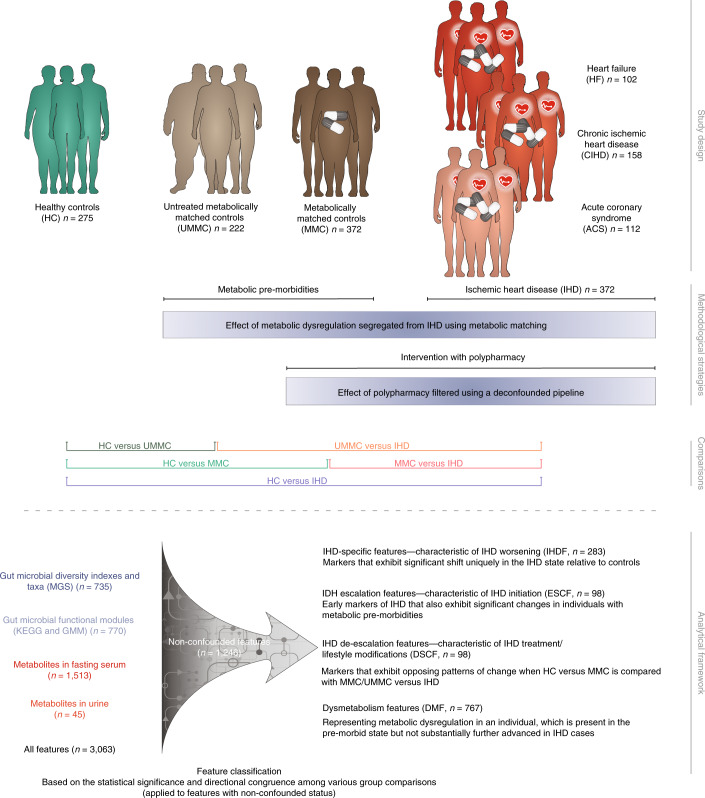
Overview of the study design. Top: the 1,241 individuals studied here are a subset of individuals from the European MetaCardis cohort, in which participants underwent deep bioclinical phenotyping combined with gut microbiome and serum and urine metabolome profiling. Participants were classified as being HCs (*n* = 275, healthy by self-report and no intake of lipid-lowering, anti-diabetic or anti-hypertensive drugs) and a combined group of patients diagnosed with IHD (*n* = 372, on various drugs). The IHD group included cases with ACS (*n* = 112), CIHD (*n* = 158) and HF (*n* = 102) due to CIHD. Two additional control groups were included: MMCs without diagnosed IHD (*n* = 372, matched on age, BMI and T2D status of the individuals with IHD, some of whom were prescribed lipid-lowering, anti-diabetic and anti-hypertensive medication but no IHD-related drugs) and untreated (non-medicated) metabolically matched non-IHD controls (UMMCs, *n* = 222, no intake of lipid-lowering, anti-diabetic, anti-hypertensive or IHD drugs). Bottom: microbiome and metabolome features were segregated into four categories, as indicated. The human icons were adapted from https://smart.servier.com/.

This study encompassed 372 individuals with IHD, including 112 with acute coronary syndrome (ACS), 158 with chronic ischemic heart disease (CIHD) and 102 with IHD and heart failure (HF). In addition, we included 275 healthy controls (HCs) matched on demographics, age and sex and 222 untreated metabolically matched controls (UMMCs)—that is, individuals with features of the metabolic syndrome and, thus, at increased risk of IHD but receiving no lipid-lowering or anti-diabetic or anti-hypertensive drugs. Finally, we included 372 controls matched with individuals with IHD in terms of T2D status and body mass index (BMI), thereafter termed metabolically matched controls (MMCs) (Fig. 1). We profiled their serum and urine metabolome (1,558 metabolites and lipids) and examined their intestinal microbiome, considering inter-individual variations in absolute fecal bacterial cell density, a factor potentially reflecting the disease state and obscuring genuine microbiome involvement^19^. Inclusion of MMC and UMMC groups allowed for the differentiation of the gut microbial and metabolomic signatures of IHD from the often-accompanied metabolic dysfunctions and related drug intake.

**Extended Data Fig. 1:**
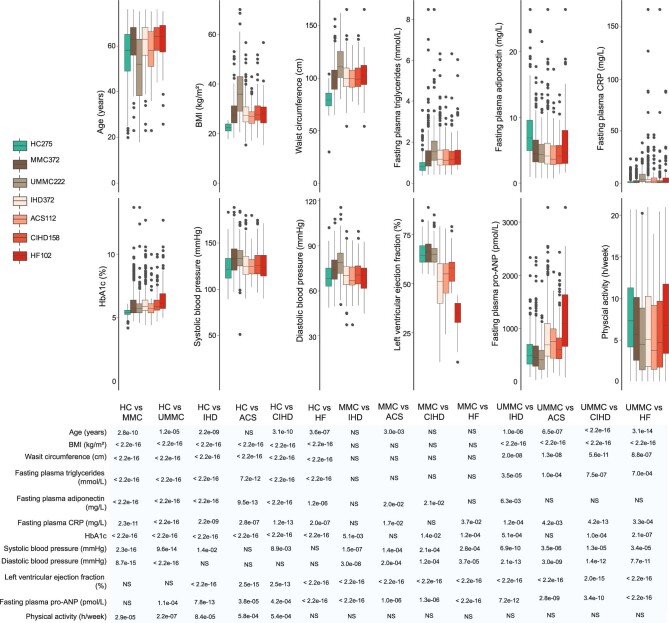
Overview of selected bio-clinical variables of the various groups. Box plots (above) representing the distribution of key bio-clinical variables in various study groups (lower line, lower quartile; medium line, median; upper line, upper quartile). Table (below) shows the two-sided MWU *P* for respective group comparisons using HC (n = 275), MMC (n = 372), UMMC (n = 222), IHD (n = 372), ACS (n = 112), CIHD (n = 158), HF (n = 102). IHD: ischemic heart disease patients, HC: healthy controls, MMC: metabolically matched controls, UMMC unmedicated metabolically matched controls, ACS: acute coronary syndrome, CIHD: chronic IHD, HF: heart failure due to CIHD, BMI: body mass index; HbA1c: glycated haemoglobin, pro-ANP: pro-atrial natriuretic peptide, MWU: Mann-Whitney U.

As expected from inclusion criteria, we found increasing CMD phenotype severity and related drug intake along the implied progression from HCs through treated and untreated metabolically matched controls (MMCs and UMMCs, respectively) to overt IHD cases (Extended Data Fig. 1 and Supplementary Tables 1–3). Despite matching for country, age, sex, BMI and T2D status, individuals with IHD remained phenotypically distinct from MMCs. They displayed increased visceral fat (*P* = 0.048), worse glycemia (HbA1c; *P* = 0.005 and fasting plasma glucose; *P* = 0.006), higher plasma concentration of liver enzymes (aspartate aminotransferase, alanine aminotransferase and γ-glutamyl transferase; *P* < 0.001) and increased prevalence and severity of hypertension (*P* < 0.001) (Supplementary Tables 1 and 2). Similarly, individuals with IHD had decreased heart contractility mirrored in reduced left ventricular ejection fraction (LVEF) and increased plasma pro-atrial natriuretic peptide (pro-ANP) levels relative to both HCs and MMCs (*P* < 0.001), which was further altered in the HF subgroup relative to ACS and CIHD (*P* < 0.001) (Extended Data Fig. 1 and Supplementary Table 2).

### Diet and physical activity variation across study groups

Diet affects microbiome composition and IHD risk^2^. We found that HCs reported healthier diets than individuals in the IHD and MMC groups, with higher values of composite metrics, such as alternative healthy eating index (aHEI^20^) (HC versus IHD, *P* < 0.001), diet diversity score (DDS^21^) (HC versus IHD, *P* = 0.001), dietary approaches to stop hypertension (DASH^22^) score (HC versus IHD, *P* = 0.013) and lower overall daily energy intake (HC versus IHD, *P* = 0.013). HCs consumed significantly less fatty animal-based food and meat and more plant-based food rich in non-digestible polysaccharides (Supplementary Table 4). They further reported higher physical activity levels (Extended Data Fig. 1), more often being in manual work and undertaking more frequent moderate to vigorous leisure time activities than individuals with IHD or MMCs (Supplementary Table 4). Some of the microbiome differences between MMCs and individuals with IHD as opposed to HCs might also reflect a less healthy lifestyle.

### Microbiome and metabolome changes related to dysmetabolism

**Fig. 2:**
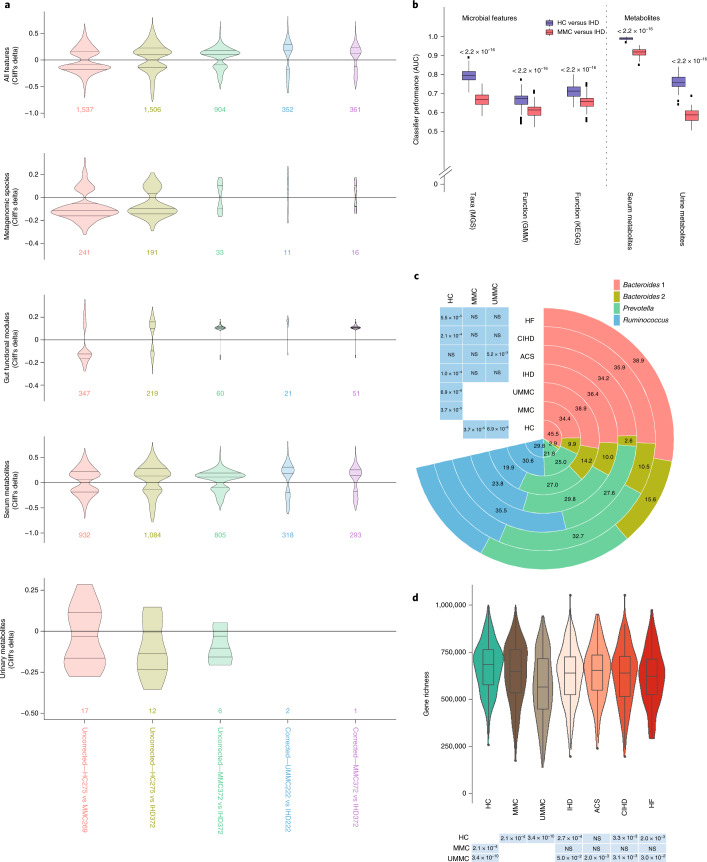
Alterations of gut microbiome and metabolome features along the natural history of IHD. **a**, Violin plots representing the distribution of significant gut microbiome and metabolome features among various group comparisons before and after data being subjected to the drug deconfounding pipeline (lower line, lower quartile; medium line, median; upper line, upper quartile). Numbers below each subplot represent total features in the respective group comparison (shown as *x* axis) that retained significance (FDR ≤ 0.1) plotted against the Cliff’s delta (*y* axis) for each set of features before (uncorrected) or after drug deconfounding (corrected). **b**, Box plots showing classifier performance comparison using HCs or MMCs as controls relative to individuals with IHD, based either on all microbial features (left) or on quantified metabolome features (right) as input (center line, median; box limits, upper and lower quartiles; whiskers, 1.5× interquartile range; points, outliers). Two-sided MWU *P* values are included for each comparison. **c**, Pie chart (right) comparing the percent (shown as numbers) distribution of four enterotypes among various study groups. Table (left) shows the chi-squared *P* value for each study group relative to the three control groups—that is, HC, MMC and UMMC. **d**, Box plots (upper) comparing gut bacterial gene richness among the indicated study groups (violin, distribution; center line, median; box limits, upper and lower quartiles; whiskers, 1.5× interquartile range; points, outliers). Table (below) shows the two-sided MWU *P* values for each study group relative to the three control groups—that is, HC, MMC and UMMC. Two-sided MWU and chi-squared tests were used for assessing the significance of group-wise comparisons in **a**, **b**, **d** and **c**, respectively, using HC (*n* = 275), MMC (*n* = 372), UMMC (*n* = 222), IHD (*n* = 372), ACS (*n* = 112), CIHD (*n* = 158) and HF (*n* = 102) groups. Multiple testing corrections were done using the Benjamini–Hochberg method, and FDR ≤ 0.1 was considered significant. NS, not significant.

**Extended Data Fig. 2:**
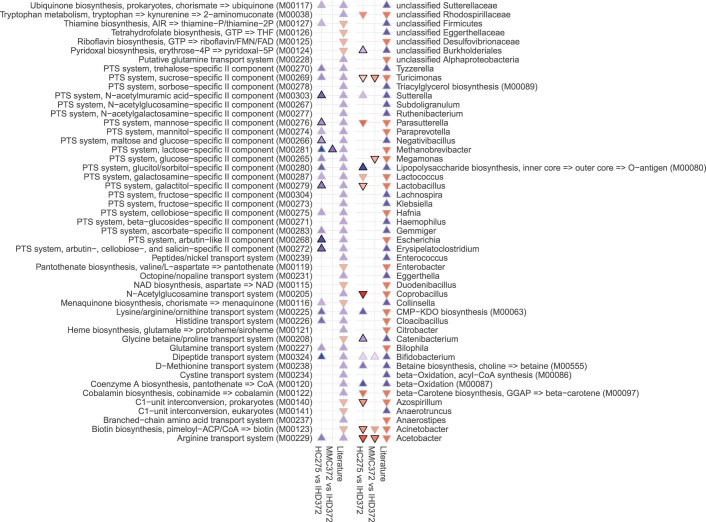
Microbiome findings from the literature. Cuneiform plot shows literature review of gut microbial taxonomic and predicted functional features reported to be associated with IHD, while highlighting their individual replication in the present MetaCardis study group either as a general dysmetabolism biomarker (seen only in case of HC versus MMC), or as an IHD biomarker (seen also in case of MMC versus IHD) (Supplementary Table 15). The literature review was performed as a keyword search in PubMed (Medline) using combinations of the words ‘microbiota’ and ‘microbiome’ with the word ‘atherosclerosis’, ‘cardiovascular disease’, ‘coronary artery disease’, ‘ischemic heart disease’, ‘myocardial infarction’, ‘acute coronary syndrome’, ‘angina pectoris’ and ‘heart failure’. Studies^61–72^ were identified that met the following criteria: 1) published during the recent 15 years, 2) reporting data from human studies with at least 15 participants, 3) using culture-independent methods for microbiota profiling and 4) evaluating the link between human microbiota and manifestations of impaired heart disease (Supplementary Table 16). Results on functional features were derived from four studies using whole-genome shotgun sequencing^68,71,72^. Results imputed from 16 S rRNA gene analyses were not included. Point marker color and size reflect MetaCardis findings (Cliff’s delta), with arrows displaying direction of effects. Literature findings are shown at a uniform effect size. Markers are shown only for features significantly different in abundance (FDR < 0.1) and have a bold border if they cannot be reduced to the confounding influence of any drug or drug combination prescribed to treat dysmetabolism. While the majority of literature findings are recaptured in our study when comparing HC and IHD, relatively fewer were found in MMC and IHD comparisons, implying them to be general markers of dysmetabolism rather than being IHD-only microbiome markers. Two-sided MWU tests were used for assessing the significance of group-wise comparisons using HC (n = 275), MMC (n = 372), UMMC (n = 222) and IHD (n = 372) groups. Multiple testing corrections were done using Benjamini-Hochberg method and FDR < = 0.1 was considered significant. IHD: ischemic heart disease patients, HC: healthy controls, MMC: metabolically matched controls, MWU: Mann-Whitney-U tests, FDR: false-discovery rate.

Both the taxonomy and functional potential of the gut microbiome as well as the metabolome differed significantly between individuals with IHD and HCs in accordance with previous reports^11–13^. Remarkably, comparing HCs to MMCs revealed even more differential features than comparing HCs to individuals with IHD (Fig. 2a and Supplementary Tables 5–8). Moreover, the discriminatory potential of microbiome and metabolome features was significantly higher between individuals with IHD and HCs than between individuals with IHD and MMCs (Fig. 2b). We recovered most previously published IHD-related gut microbiome findings (Extended Data Fig. 2 and Supplementary Tables 15 and 16), primarily by contrasting HCs and individuals with IHD. However, most were already significant in MMC versus HC comparisons, suggesting that previous studies might have erroneously reported dysmetabolism features as bona fide IHD features. These might contribute to increased risk of IHD, but our analyses indicate that they are not specific for IHD.

**Extended Data Fig. 3:**
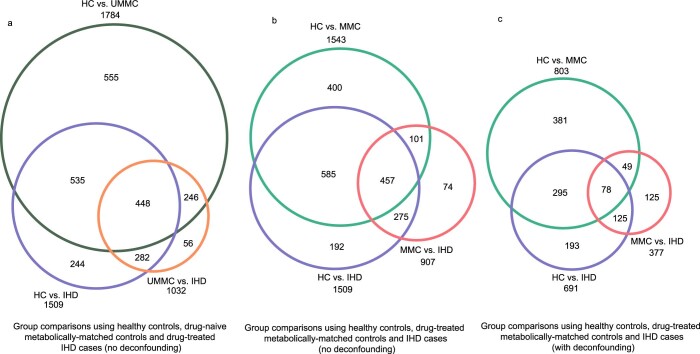
Distribution of differential features among various group comparisons pre- and post- deconfounding. (a) Venn diagrams showing the comparative shift in the number of gut microbiome and metabolome features that remain differentially abundant (FDR < 0.1) in various group comparisons when healthy individuals (HC) and drug-treated IHD cases are compared to untreated metabolically matched controls (UMMC) or (b) drug-treated metabolically matched controls (MMC) without any adjustments for potential confounders followed by (c) drug-deconfounding. Two-sided MWU tests were used for assessing the significance of group-wise comparisons using HC (n = 275), MMC (n = 372), UMMC (n = 222) and IHD (n = 372) groups. Multiple testing corrections were done using Benjamini-Hochberg method and FDR < = 0.1 was considered significant. IHD: ischemic heart disease patients, MWU: Mann-Whitney-U tests, FDR: false-discovery rate.

At higher microbiome architecture levels, there was a significant shift from the *Bacteroides* 1 and *Ruminococcus* enterotypes toward the low bacterial cell-count-associated *Bacteroides* 2 as disease worsened^23^ (Fig. 2c). These findings mirror significant loss of microbial gene richness (Fig. 2d) and absolute gut bacterial cell load (that is, microbial load) in both MMCs and individuals with IHD to HCs. In contrast, no differences were found when individuals with IHD were compared to MMCs (Supplementary Table 5). Bacterial gene depletion and *Bacteroides* 2 prevalence were even more exacerbated in UMMCs, possibly due to drugs not yet being prescribed and the presence of a more obese phenotype in this group^24^. Consistently, the total number of gut microbiome and metabolome features significantly differential in abundance was higher when HCs were compared to UMMCs relative to MMCs (Extended Data Fig. 3).

### Microbiome and metabolome signatures of IHD

**Fig. 3:**
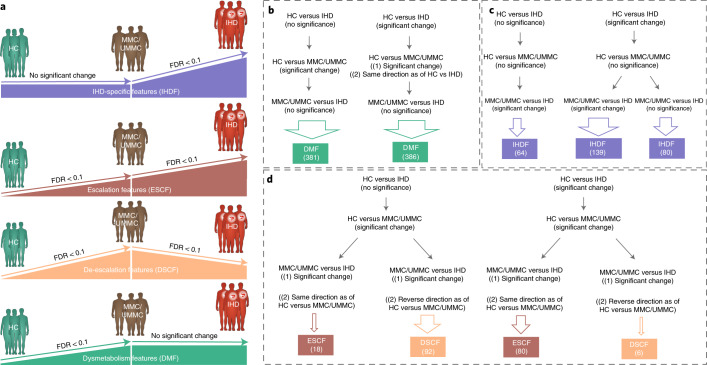
Approach used for categorization of microbiome and metabolome features in the cross-sectional study. **a**–**d**, Gut microbiome and plasma and urine metabolome features that exhibited a statistically significant shift uniquely when treated MMCs, untreated UMMCs and treated individuals with IHD were compared with HCs were categorized as DMFs (**a**,**b**) as these features exhibited significant alterations in association with metabolic syndrome (that is, obesity and T2D) and not IHD per se. In contrast, gut microbiome and plasma and urine metabolome features that exhibited a significant change when either MMCs or UMMCs were compared with individuals with IHD were categorized as IHDFs. In addition, features exhibiting a significant change in individuals with IHD relative to HCs were categorized as IHDFs when they exhibited a simultaneous significant shift in individuals with IHD relative to MMCs or UMMCs (**a**,**c**). Next, we considered the natural trajectory of IHD in two stages—that is, HCs versus MMCs or UMMCs (representing the dysmetabolism stage) and MMCs or UMMCs versus individuals with IHD (representing the IHD stage). Features exhibiting a significant change under both dysmetabolic and IHD stages and in the same direction (representing disease progression) were thus labeled as ESCFs (**a**,**d**), whereas those exhibiting a significant change in the reverse direction (representing disease stabilization) were labeled as DSCFs (**a**,**d**). Our approach evaluated every feature across all group comparisons using the criteria of (1) non-confounded status (that is, feature cannot be confounded by any tested host variables, including drug treatment); (2) significance status (that is, feature has to exhibit FDR < 0.1 for respective group comparison); and (3) a directional alignment status (that is, direction of change when disease stages are considered) for categorization as DMF (**b**), IHDF (**c**), ESCF or DSCF (**d**). (See Extended Data Fig. 4 and Methods for more details.) The arrow size further reflects the number of features identified by each route for respective categorization: 767 DMFs, 283 IHDFs and 98 each of ESCFs and DSCFs were identified. Two-sided MWU was used for assessing the significance of group-wise comparisons using HC (*n* = 275), MMC (*n* = 372), UMMC (*n* = 222) and IHD (*n* = 372) groups. Multiple testing corrections were done using the Benjamini–Hochberg method, and FDR ≤ 0.1 was considered significant. The human icons were adapted from https://smart.servier.com/.

**Fig. 4:**
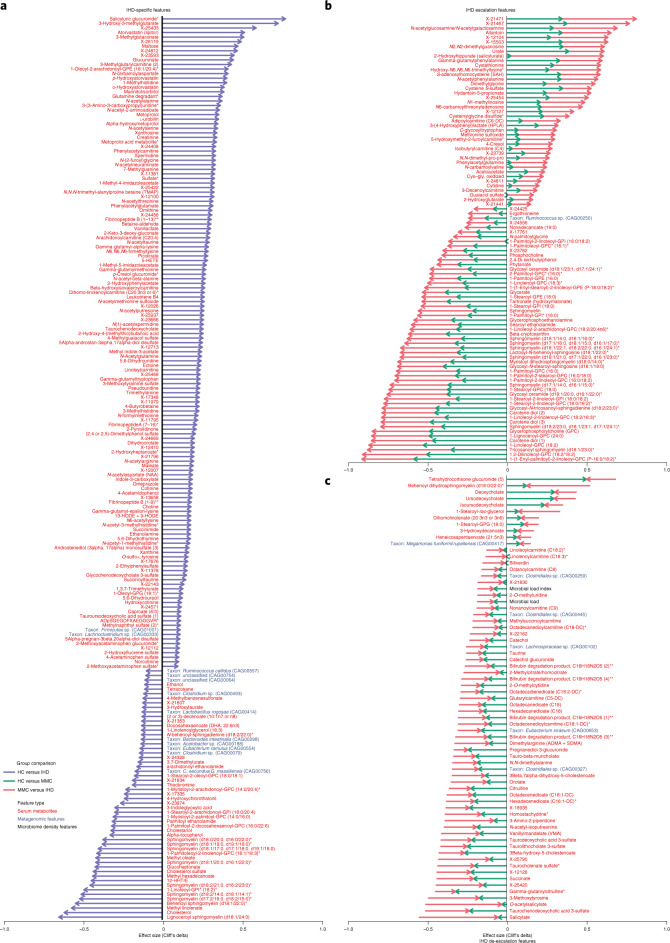
Microbiome and metabolome features linked with IHD and its dysmetabolic pre-morbidities. Using the categorization scheme described in Fig. 3 and Extended Data Fig. 4, gut microbiome and metabolome markers were categorized as DMFs, IHDFs, ESCFs or DSCFs, of which IHDFs (**a**), ESCFs (**b**) and DSCFs (**c**) are displayed here. In each panel, arrow length shows effect sizes (Cliff’s delta) for respective group comparisons. Cliff’s delta for HC versus IHD comparisons are displayed for IHDFs (**a**), whereas Cliff’s delta for both HC versus MMC and MMC versus IHD are displayed for ESCFs (**b**) and DSCFs (**c**), with arrowhead pointing to the direction of change. Only features exhibiting an absolute effect size greater than 0.1 are displayed, inclusive of serum metabolites, metagenomic species and microbial density indices (see Supplementary Table 17 for a description of effect sizes and confounding status). Two-sided MWU was used for assessing the significance of group-wise comparisons using HC (*n* = 275), MMC (*n* = 372), UMMC (*n* = 222) and IHD (*n* = 372) groups. Multiple testing corrections were done using the Benjamini–Hochberg method, and FDR ≤ 0.1 was considered significant. *The metabolite was not validated by an internal standard but confirmed with great confidence according to information from Metabolon (Methods) who performed the analysis. **An internal standard for the metabolite was not available but was confirmed with reasonable confidence according to information from Metabolon (Methods) who performed the analysis.

**Extended Data Fig. 4:**
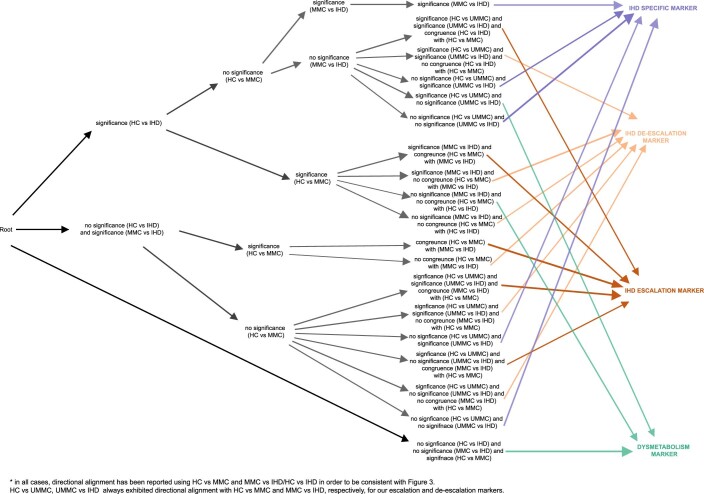
Operational classification of microbiome and metabolome features from the perspective of IHD pathology. A classification tree was constructed based on significance and alignment of effect size and directionality of microbiome and metabolome features in the various group comparisons leading to the identification of: Features that reflect metabolic dysregulation in the individual but are not associated with diagnosed IHD: **dysmetabolism features (DMF)**. Features that are significantly associated with IHD but are also significantly altered in metabolically dysregulated individuals in the same direction; we suggest that these features are early markers of IHD pathogenesis in individuals with metabolic dysregulation: **IHD escalation features (ESCF)**. Features that are significantly associated with IHD but are also significantly altered in metabolically dysregulated individuals in the reverse direction; we suggest that these features are early markers of IHD seen in metabolically dysregulated individuals. However, they exhibit reversibility. This may plausibly be due to 1) long-term drug-treatment and improvement in overall lifestyle of the IHD individuals, 2) a compensatory response to the initiation of disease or 3) a trajectory-associated differential response to disease development. We propose that some of these features contribute to the stabilization of IHD and dysmetabolism and we coin those **IHD de-escalation features (DSCF)**. **IHD-specific features (IHDF)** that achieve a significant shift only under IHD diagnoses. Two-sided MWU tests were used for assessing the significance of group-wise comparisons using HC (n = 275), MMC (n = 372), UMMC (n = 222), IHD (n = 372), ACS (n = 112), CIHD (n = 158), HF (n = 102) groups. Multiple testing corrections were done using Benjamini-Hochberg method and FDR < = 0.1 was considered significant. HC: healthy controls, MMC: metabolically matched controls, UMMC unmedicated metabolically matched controls, IHD: ischemic heart disease, ACS: acute coronary syndrome, CIHD: chronic IHD, HF: heart failure due to IHD, MWU: Mann-Whitney U, FDR: false-discovery rate.

We consider the identification of genuine microbiome and metabolome signatures of IHD—that is, disease features not better explained as indirect associations via drugs and demographics—to be a major contribution of our study. Additionally, we further differentiate IHD features from their metabolic morbidities by categorizing them according to their signatures among the various group comparisons across the CMD spectrum, focusing qualitatively on condition specificity and quantitatively on effect size (Fig. 3 and Extended Data Fig. 4). We identify features as being specific to dysmetabolism (Fig. 3a,b) or IHD (Fig. 3a,c) by exhibiting a significant change only under the respective condition—that is, HCs versus MMCs/UMMCs for dysmetabolic features (DMFs) or MMC/UMMCs versus individuals with IHD for IHD-specific features (IHDFs). Additionally, we identify features based on whether they exhibit a typical shift in effect size in both dysmetabolism and IHD, either maintaining it in the same direction from dysmetabolism to IHD—that is, escalation features (ESCFs)—or, on the contrary, in the opposite direction—that is, de-escalation features (DSCFs) (Fig. 3a,d). Specifically, ESCFs represent early markers of IHD that continue to increase/decrease during metabolic morbidity (that is, HCs versus MMCs/UMMCs) to overt IHD (that is, MMCs/UMMCs versus individuals with IHD) (Fig. 4b). In contrast, DSCFs exhibit a reverse pattern of shift when considering the effect sizes between HCs versus MMCs/UMMCs and MMCs/UMMCs versus individuals with IHD (Fig. 4c). In brief, for features already aberrant in MMCs, DSCFs represent those being restored toward HC levels in diagnosed and treated IHD, plausibly associated to disease stabilization.

Most significant IHD-associated features were categorized as primarily indicators of general dysmetabolism rather than specific to IHD, whereas next in order of frequency were features specific to IHD and then de-escalation and escalation features (Figs. 1 and 3, Supplementary Fig. 1 and Supplementary Table 17). This pattern remained largely valid also when the three IHD subtypes were considered separately (Supplementary Fig. 2), in line with our observation of a major shift in gut microbiome and metabolome during the dysmetabolic stage before IHD diagnosis.

**Fig. 5:**
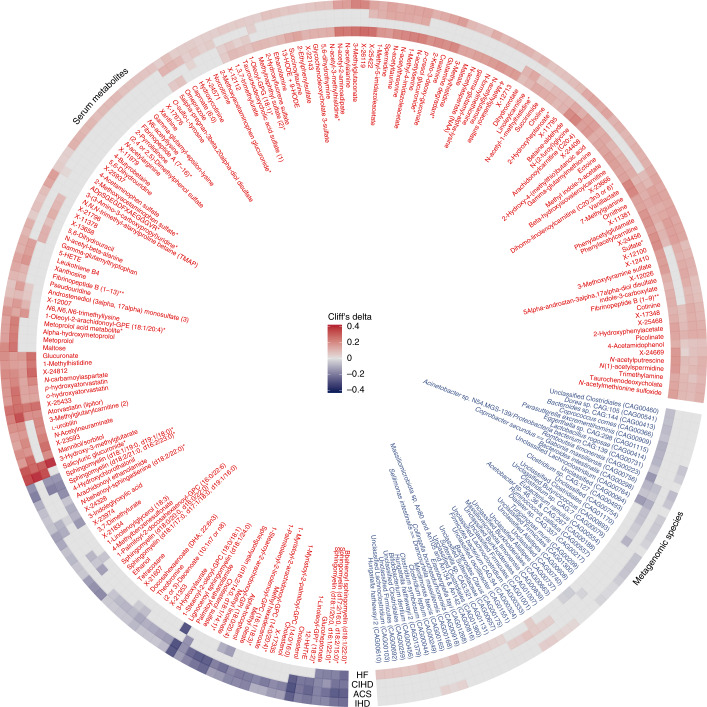
Metabolome and microbiome features altered uniquely in IHD and its subtypes. Circle plot shows gut microbial species and serum metabolites that were categorized as being specific to IHD or to its subtypes—ACS, CIHD and HF due to CIHD as per our categorization scheme shown in Fig. 3 and Extended Data Fig. 4. Each layer shows effect sizes (Cliff’s delta) for individual features that were either enriched or depleted in cases (IHD or its subtypes) versus HCs (see also Supplementary Table 17 for all features listed as being specific to IHD and its subtypes). Only features exhibiting absolute effect sizes greater than 0.1 for HC versus IHD are displayed. *The metabolite was not validated by an internal standard but confirmed with great confidence according to information from Metabolon (Methods) who performed the analysis. **An internal standard for the metabolite was not available but was confirmed with reasonable confidence according to information from Metabolon (Methods) who performed the analysis.

**Extended Data Fig. 5:**
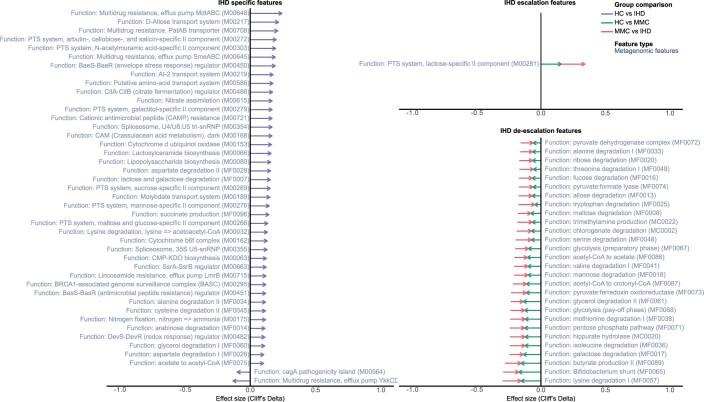
Gut microbial functional features categorization. Gut microbial functional features (GMM and KEGG modules) categorized as escalation-, de-escalation-, and IHD-specific biomarkers when features classification scheme (as shown in Fig. 3, Extended Data Fig. 4 and described in supplementary methods) was applied to various group comparisons involving HC, MMC and IHD subjects. HC: healthy controls, MMC: metabolically matched controls, IHD: ischemic heart disease.

Of 121 species that were markers of dysmetabolism (that is, DMFs) (Supplementary Table 17), an overwhelming majority (85%) were depleted in IHD, paralleling observations for the ACS cases analyzed in the companion paper^25^. Twenty-three species were IHD-specific markers (Figs. 4a and 5), with a similar trend toward depletion in patients (65%). They included three proteobacteria—*Acinetobacter, Turcimonas* and *Acetobacter*—that were previously reported depleted in IHD (Extended Data Fig. 2). Among eight species enriched in IHD, two were Betaproteobacteria of the Burkholderiales order. Interestingly, *Burkolderia pseudomallei* is reported as a possible cause of endocarditis^26^. A single species, an uncharacterized Ruminococcus depleted in IHD, was an IHD escalation marker (Fig. 4b); ruminococci include butyrate producers, and their depletion might contribute to the reduced production potential of short-chain fatty acids (SCFAs) in IHD. Six species were de-escalation markers (Fig. 4c); they belonged to the Clostridiales order, and all but one, *Eubacterium siraeum*, were unclassified at species or even genus taxonomic level. *Eubacterium* was previously reported to be depleted in atherosclerosis (Fig. 4 and Supplementary Table 17). In contrast, microbiome functions (gut metabolic modules (GMMs) and Kyoto Encyclopedia of Genes and Genomes (KEGG) modules) were mostly enriched in IHD (Extended Data Fig. 5).

In parallel, the metabolome reporting most of IHD-specific markers showed a marked enrichment, with only 50 of 203 IHD-specific markers (25%) being depleted in IHD relative to HC (Fig. 4 and Supplementary Table 17). We identified enrichment of a range of IHD-specific metabolites, including intermediaries of the choline and carnitine pathways quantified by ultra-performance liquid chromatography with tandem mass spectrometry (UPLC–MS/MS)—that is, choline, betaine-aldehyde, 4-butyrobetaine, linoleylcarnitine and trimethylamine (TMA), the precursor of trimethylamine *N*-oxide (TMAO), which is known to modulate IHD risk^27^. Other such carnitine metabolites included medium-chain and long-chain fatty acyl carnitines, suggesting an increase in transport into the mitochondria through the carnitine shuttle, typically for β-oxidation. In particular, microbial aromatic acids, such as phenylacetate, reported to be inversely associated with species-level genome bin (SGB) 4712 in the companion paper^25^, or benzoate, follow a similar process, producing phenylacetatylcarnitine or benzoylcarnitine. They undergo conjugation with amino acids to form, for instance, phenylacetylglutamate or hippurate^28^, of which both phenylacetylcarnitine and phenylacetylglutamate are IHD-specific markers in our study (Fig. 4a).

**Extended Data Fig. 6:**
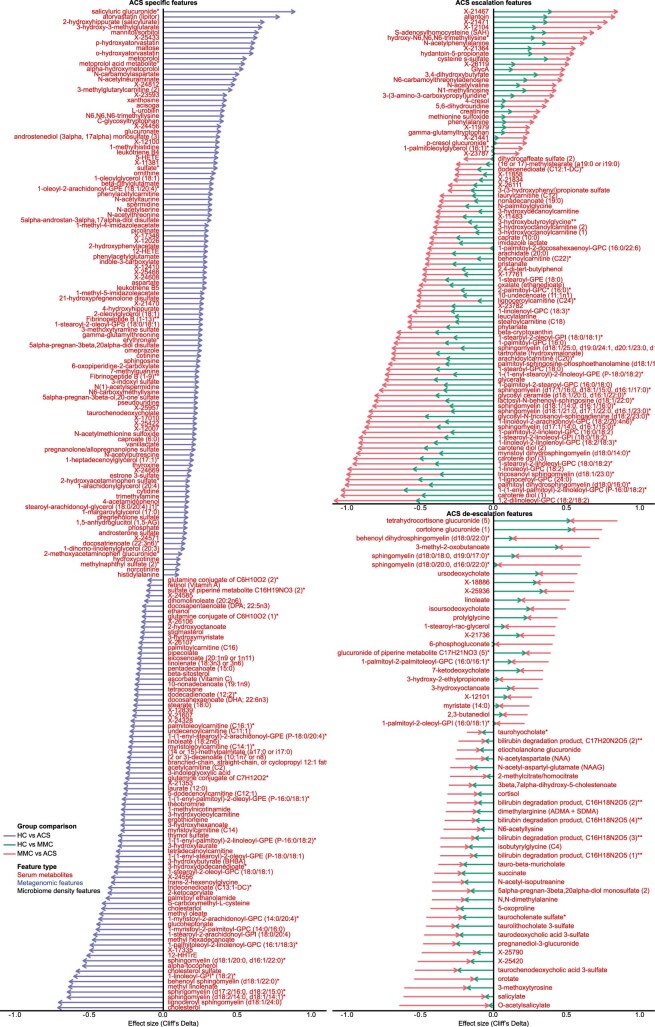
Features categorization for ACS subgroup. Microbiome and metabolome features categorized as escalation-, de-escalation-, and ACS-specific biomarkers when features classification scheme (as shown in Fig. 3, Extended Data Fig. 4 and described in supplementary methods) was applied to various group comparisons involving HC, MMC and ACS groups. HC: healthy controls, MMC: metabolically matched controls, ACS: acute coronary syndrome, ESCF: escalation features, DSCF: De-escalation features. Gut microbiome features included taxonomic (prefix: Taxon) and microbiome density indices, whereas metabolome features included serum and urinary metabolites. Only features exhibiting absolute effect size > 0.1 are displayed whereas the full list is given in Supplementary Table 17).

**Extended Data Fig. 7:**
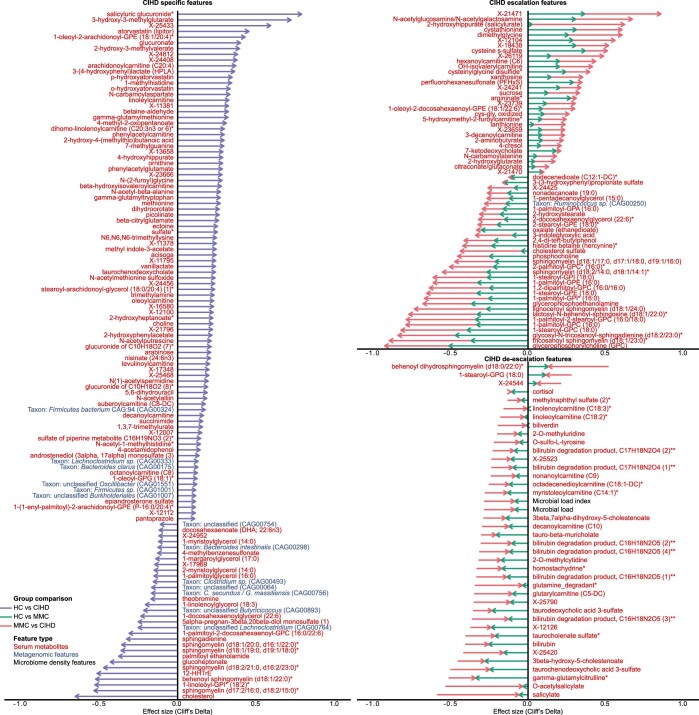
Features categorization for CIHD subgroup. Microbiome and metabolome features categorized as escalation-, de-escalation- and CIHD-specific biomarkers when features classification scheme (as shown in Fig. 3, Extended Data Fig. 4 and described in supplementary methods) was applied to various group comparisons involving HC, MMC and CIHD groups. HC: healthy controls, MMC: metabolically matched controls, CIHD: chronic IHD. ESCF: escalation features, DSCF: De-escalation features. Gut microbiome features included both taxonomic (prefix: Taxon) and microbiome density indices, whereas metabolome features included serum and urinary metabolites. Only features exhibiting absolute effect size > 0.1 are displayed whereas the full list is given in Supplementary Table 17).

**Extended Data Fig. 8:**
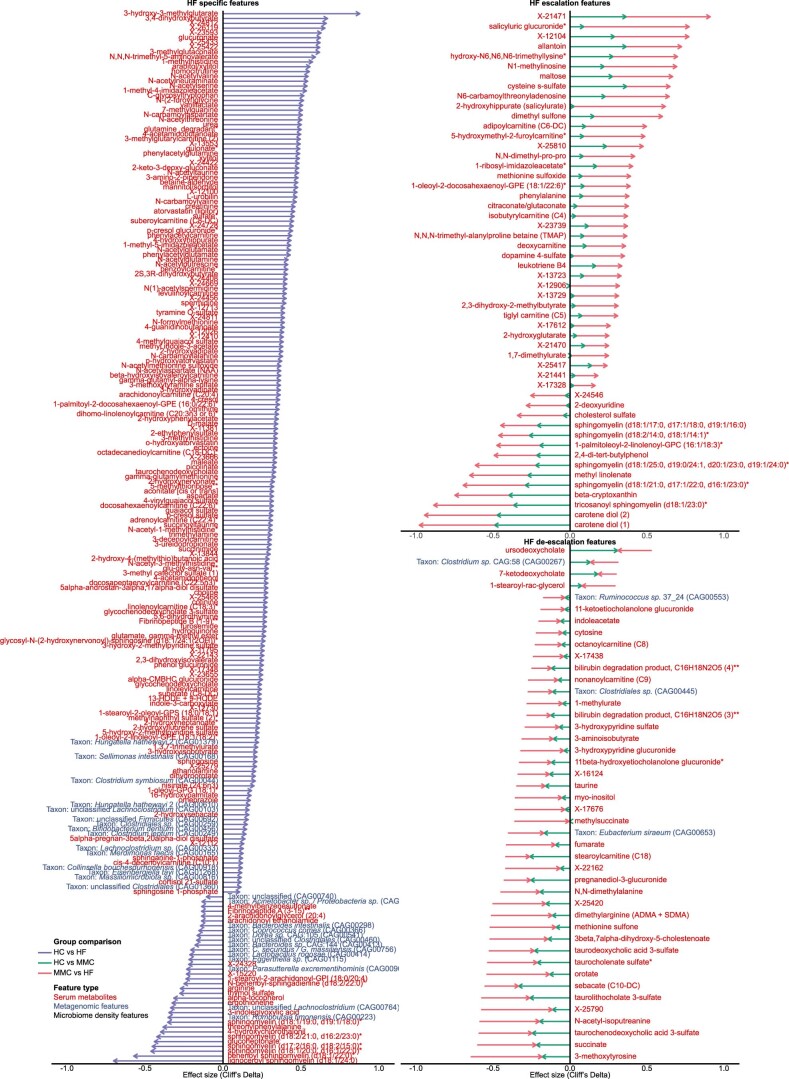
Features categorization for HF subgroup. Microbiome and metabolome features categorized as escalation-, de-escalation- and HF-specific biomarkers when features classification scheme (as shown in Fig. 3, Extended Data Fig. 4 and described in supplementary methods) was applied to various group comparisons involving HC, MMC and HF groups. HC: healthy controls, MMC: metabolically matched controls, HF: heart failure due to CIHD. ESCF: escalation features, DSCF: De-escalation features. Gut microbiome features included both taxonomic (prefix: Taxon) and microbiome density indices, whereas metabolome features included serum and urinary metabolites. Only features exhibiting absolute effect size > 0.1 are displayed whereas the full list is given in Supplementary Table 17**)**.

Along the same lines, we observed an increase in pro-inflammatory lipids derived from arachidonic acid (C20:4) starting with arachidonoylcarnitine, 5-hydroxyeicosatetraenoic acid (5-HETE) as well as leukotriene B4 and 9-/13- hydroxyoctadecadienoic acid (9-HODE/13-HODE), which are known mediators of inflammation and atherogenesis^29,30^. In contrast, fatty acid methyl esters, including methyl hexadecanoate, methyl linolenate and methyl oleate, along with alpha-tocopherol, known for vasoprotective^31^ and antioxidant properties^32^, respectively, were among the top metabolites whose depletion constitutes markers specific for IHD (Fig. 4a). Notably, similar patterns remained in IHD subtype-specific analyses (Extended Data Figs. 6–8 and Supplementary Table 17).

Most IHD escalation features represented by the metabolome exhibited an initial depletion upon dysmetabolism, which continued after IHD diagnosis (Fig. 4b and Supplementary Table 17). Besides several complex phospholipids, including sphingomyelins and glycerophospholipids, several carotenoids (for example, carotene diols and β-cryptoxanthin) and ergothioneine, which are known to improve cardiovascular health, exhibited the above depletion pattern, whereas glutathione metabolism and markers of oxidative stress (for example, cystathionine and cys-gly oxidized) instead escalated. Ergothioneine, in particular, has been associated with reduced cardiovascular and overall mortality^33^ and was also identified as a key metabolite exhibiting a positive correlation with SGB 4712 (that is, both SGB 4712 and ergothioneine exhibiting depletion) in ACS cases relative to controls in the companion paper^25^. Consistently, in the present study, a reduction in circulating levels of ergothioneine was also observed in individuals with ACS and HF relative to HCs (Supplementary Table 17).

In contrast, 4-cresol exhibited an enrichment pattern from dysmetabolism to IHD (Fig. 4b). 4-cresol is a bacterial product of colonic fermentation of phenylalanine and tyrosine and a precursor for uremic toxin 4-cresylsulfate. Similarly, phenylacetylglutamine, another uremic toxin derived from microbial phenylacetate and that acts through adrenergic receptors^34^, showed an enrichment pattern from dysmetabolism to IHD. It was also shown (by ref. ^25^) to be inversely associated with SGB 4712. The findings implicate these metabolites as key targets for early intervention. 4-Cresol, in particular, has been found in lower concentrations in the blood of vegetarians than of omnivores^35^; it has also been shown to inhibit colonocyte oxygen consumption^36^ and to be reduced once fat intake is curbed^37^. In our study, this compound appeared as an ACS and CIHD escalation feature, and it was also one of the top markers specifically enriched in the blood of individuals with HF, likely related to its role in uremia^38^, with dysregulation of fluid homeostasis being a key feature of HF (Extended Data Fig. 7). Interestingly, we also observe in another MetaCardis study that 4-cresol plays a causal role in the gut microbiome–kidney–heart axis in HCs, culminating in increased pro-ANP levels (Chechi et al., in revision).

Most DSCFs (89% and 100% for metabolites and predicted microbiome functions, respectively) exhibited the pattern of initial depletion at the stage of dysmetabolism but an apparent reversal at the stage of treated IHD (Fig. 4c). For instance, *O*-acetylsalicylate, the active component in aspirin, appeared as an archetypal DSCF putatively due to patient treatment compliance in IHD. Similarly, several catecholamine intermediates and end-products, bilirubin products, bile acids and odd-chain lipids with bacterial origin were identified as DSCFs. Moreover, TMA production (MC0022) and butyrate production II (MF0089) as gut microbial functional features exhibited a depletion at the dysmetabolism stage but an apparent restoration at the IHD stage (Extended Data Fig. 5). Overall, these observations might point toward a responsiveness of both microbiome and metabolome features to long-term multifactorial treatment, plausibly contributing to stabilization of IHD. In addition, achieving a stabilized IHD state appeared to involve restoring lost gut microbial cell density (Fig. 4c) alongside a capacity to degrade BCAAs and galactose while restoring lost capacity for butyrate and acetate production (Extended Data Fig. 5).

### Microbiome and metabolome markers of IHD sub-phenotypes

**Extended Data Fig. 9:**
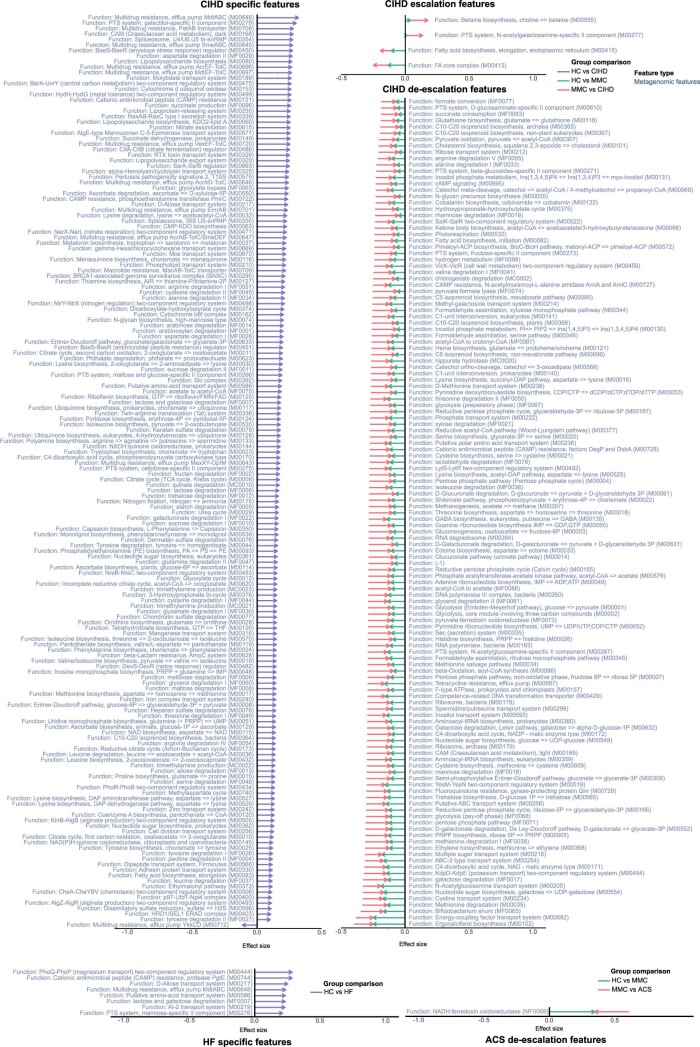
Gut microbial functional features categorization for IHD subgroups. Microbial functional features (GMM and KEG modules) categorized as escalation-, de-escalation- and subtype-specific biomarkers when features classification scheme (as shown in Fig. 3, Extended Data Fig. 4 and described in supplementary methods) was applied to various group comparisons involving HC, MMC and IHD subgroups (that is, ACS, CIHD and HF). HC: healthy controls, MMC: metabolically matched controls, ACS: acute coronary syndrome, CIHD: chronic IHD, HF: heart failure due to CIHD. Only features exhibiting absolute effect size > 0.1 are displayed whereas the full list is given in Supplementary Table 17).

Detailed analysis of ACS-, CIHD- and IHD-caused HF groups provided more granularity for relative shifts in microbiome and metabolome features (Fig. 5, Extended Data Figs. 6–9 and Supplementary Table 17).

The total number of features typical for each IHD subgroup compared to controls was highest for CIHD, followed by HF and ACS. CIHD exhibited the most differential changes in the gut microbiome functional potentials (Extended Data Fig. 9), whereas ACS exhibited predominantly differential changes in metabolome features (Fig. 5, Extended Data Figs. 6–9 and Supplementary Table 17).

Most (69%) of the dysmetabolism-linked species found by IHD versus HC comparisons were also present in comparisons of IHD subgroups versus HC, suggesting that the major disruption of the microbiome, which appears to be related to metabolic dysfunction, might persist throughout the various stages of IHD.

Strikingly, for the ACS subgroup, besides the 91 dysmetabolism-related species, no other species markers (ACS-specific, ESCF-related or DSCF-related) were found (Supplementary Table 17). In the same ACS group, the pattern was very different for serum metabolites where only 55% of markers were related to dysmetabolism, whereas 25% were ACS-specific (Supplementary Table 17). We, thus, observed the acute disease phase being characterized by microbiome alterations almost exclusively related to dysmetabolism, presumably accumulating during the long prodromal stage, as well as host metabolome perturbations unrelated to dysmetabolism, presumably beginning only shortly before the ACS event. It is tempting to suggest that the conjunction of the two might be conducive to some of the decompensation observed in ACS.

When considering the metabolome markers specific to ACS, eight of the top ten metabolites were drug analytes or drug metabolites, related to aspirin, metroprolol and atorvastatin. There was also an increase in pro-inflammatory metabolites such as 5- and 12-hydroxyeicosatetraenoic acid (HETE), leukotriene B4 and B5, as well as products of microbial–host phenylalanine co-metabolism (phenylacetylcarnitine, phenylacetylglutamate and 2-hydroxyphenylacetate), followed by indoxylsulfate and TMA, which is consistent with the identified overall IHD-specific signature. Likewise, some of the ACS-specific depleted metabolites were also less abundant in IHD, including health beneficial metabolites such as alpha-tocopherol, ergothioneine, methyl oleate and methyl hexadecanoate (Extended Data Fig. 6 and Supplementary Table 17).

In contrast to the findings in ACS, 19 and 31 specific species markers were found for CIHD and HF, respectively, indicating additional microbiome changes in the chronic phases of IHD. Noticeably, these changes affected genera represented by only a few species: eight of 14 depleted and 11 of 17 enriched species in HF cases, respectively, belonged to genera represented by no more than six species (*P* = 2.9 × 10^−5^) as estimated by the number of species belonging to different genera found in our study (Extended Data Figs. 7 and 8 and Supplementary Table 17).

Most CIHD-specific features was enriched in cases over controls (Extended Data Figs. 7 and 9 and Supplementary Table 17). This was particularly the case for microbiome functional potentials for amino acid biosynthesis, including BCAA (KEGG modules M00019, M00570 and M00432), methionine (KEGG module M00017) and lysine (KEGG module M00030) (Extended Data Fig. 9). Similarly, enhanced degradation of aromatic amino acids phenylalanine and tyrosine (GMM modules MF0027 and MF0026) was reflected by increased abundance of phenylacetate metabolites (phenylacetylcarnitine and phenylacetylglutamate). We also observed increased abundance of methionine and two of its metabolites (*N*-acetylmethionine sulfoxide and γ-glutamylmethionine), which are known to be associated with cardiovascular phenotypes^39^. Of interest, the gut microbiome-derived l-methionine biosynthesis pathway was recently directly associated with atherosclerotic plaque burden and enhanced metabolic risk score for cardiovascular disease^18^, whereas l-methionine sulfoxide as a product of protein methionine oxidation might influence thrombosis and vascular function^40^ (Extended Data Figs. 7 and 9 and Supplementary Table 17). In addition, the abundance of multiple UPLC–MS/MS-quantified carnitines, including decanoylcarnitine and oleoylcarnitine, was elevated in CIHD.

Some metabolite features also exhibited HF specificity with an enrichment of 4-cresol, 4-cresyl sulfate (also called *p*-cresol sulfate), 4-cresylglucuronide (also called *p*-cresol glucuronide), choline and TMA as well as several carnitines (3-methylglutarylcarnitine, suberoylcarnitine (C8), octadecanedioylcarnitine (C18) and levulinoylcarnitine, including microbiome-derived carnitines (benzoylcarnitine and phenylacetylcarnitine)). In contrast, metabolites, such as alpha-tocopherol, ergothioneine and 3-indoleglyoxylic acid, exhibited HF-specific depletion (Extended Data Fig. 8 and Supplementary Table 17). These findings point toward altered fatty acid metabolism, which is known to play a crucial role in HF pathogenesis^41^.

### Classification of participants into clinical subgroups

**Extended Data Fig. 10:**
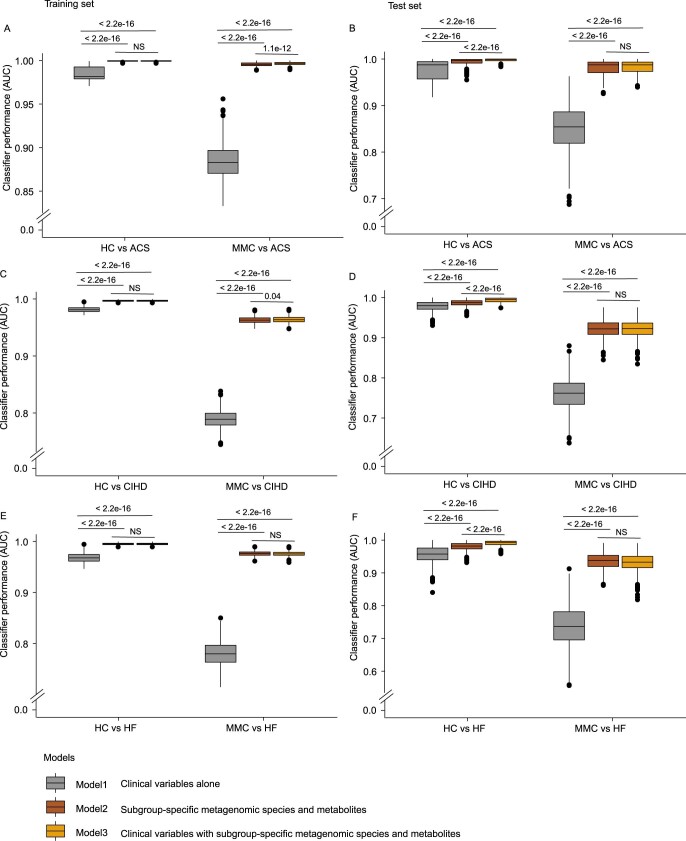
Discriminatory potential IHD subtype-specific features. We compared clinical variables assessed for risk prediction in the companion paper^25^ (Model 1) with our IHD subgroup-specific gut microbiome and metabolomic features (Model 2) and a combination of the two (Model 3) for their discriminatory potentials using orthogonal partial least squares- discriminant analysis (O-PLS-DA; ropls r package). Model 1 included ten variables (*that is* age, sex, body mass index, waist circumference, hip circumference, waist to hip ratio, systolic blood pressure, diastolic blood pressure, glycated haemoglobin (factored as > 5.7, 5.7-6.4 and < 6.4 mmol/l) and smoking status). Model 2 included each IHD subgroup-specific metagenomic species and fasting serum metabolites. Model 3 involved a combination of model 1 and 2 variables. OPLS-DA models were trained on 70% of the subgroup specific sample and then tested in 30% of the remaining subgroup sample using 1000 iterations of random sampling (bootstrapping). Boxplots represent the distribution (center line, median; box limits, upper and lower quartiles; whiskers, 1.5× interquartile range; points, outliers) of area under the receiver operating characteristic (ROC) curves derived from 1000 bootstraps based on these models in the training set (A) and test set (B) using both healthy controls (HC, n = 275) and metabolically matched controls (MMC, n = 372) relative to the IHD subtype cases (ACS, n = 112, CIHD n = 158 and HF n = 102). Models were compared using Kruskal-Wallis test and Dunn’s pairwise multiple comparisons post hoc testing with Bonferroni correction. Dunn’s test *P* are shown for each comparison. As expected, the model performance improves significantly for model 2 and 3 relative to model 1, respectively, when either HC or MMCs are used as controls for IHD cases in test samples. HC: healthy controls, MMC: metabolically matched controls, IHD: ischaemic heart disease. ACS: acute coronary syndrome, CIHD: chronic IHD, HF: heart failure due to CIHD.

Robustness of our microbiome and metabolome signatures was evaluated by comparing the performance of orthogonal partial least squares discriminant analysis (O-PLS-DA) models for classifying ACS (*n* = 112), CIHD (*n* = 158) and HF (*n* = 102) relative to HC (*n* = 275) and MMC (*n* = 372) (Extended Data Fig. 10). Classification was based on (1) clinical markers routinely assessed during IHD diagnosis; (2) deconfounded microbiome and metabolome markers specific for each IHD subtype identified in the current study; and (3) a combination of the two. Models were built by randomly splitting our MetaCardis study population into groups of 70% and 30%, respectively, and using the former for training and the latter for testing; the process was iterated 1,000 times to minimize overfitting. The performance of the specific -omics markers on the testing sets yielded area under the curve (AUC) values greater than 0.7 in all cases and was systematically higher than that of clinical markers only, in particular for classification relative to the MMC group. Combination of the two marker types did not improve classification relative to MMC and only marginally improved classification relative to HC (Extended Data Fig. 10).

**Fig. 6:**
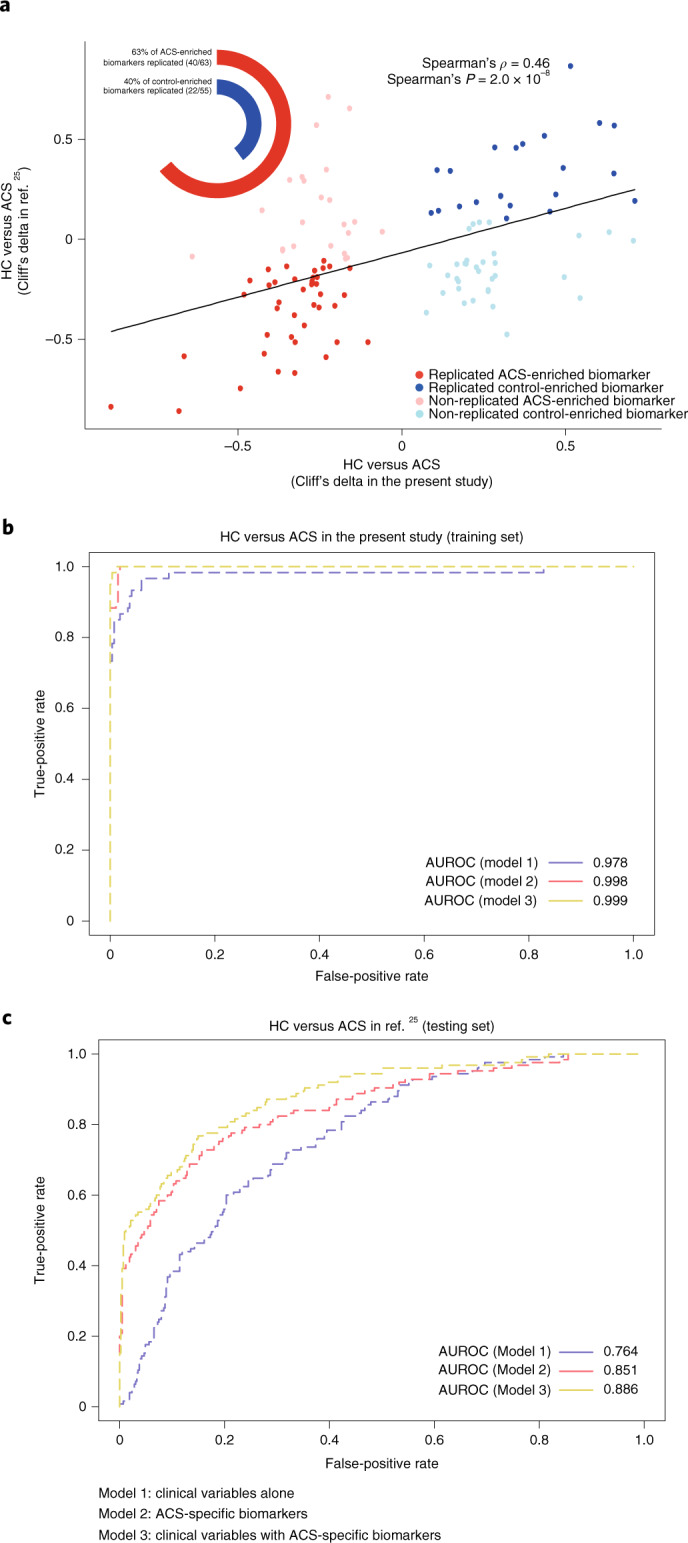
Validation of markers for ACS. **a**–**c**, For the gut microbial and plasma metabolome features common to both MetaCardis and Israeli cohorts, a Spearman correlation analysis (**a**) was conducted between the effect sizes (Cliff’s delta) for HC versus ACS comparison in each study after recalculating Cliff’s deltas in the Israeli population. Next, ROC curves depicting the classifier performance (AUROC) of five-fold cross-validated O-PLS-DA models based on the overlapped set of ACS biomarkers in three settings are shown for MetaCardis as the training population (**b**) and Israeli cohort as the test population (**c**). Model 1 included nine clinical ACS risk variables—that is, age, sex, BMI, systolic blood pressure, diastolic blood pressure, glycated hemoglobin (factored as >5.7, 5.7–6.4 and <6.4 mmol l^−1^), smoking status, fasting total cholesterol and HDL cholesterol (mmol l^−1^). Model 2 included ACS-specific biomarkers identified in our study that were also found in ref. ^25^ (118 variables), whereas model 3 involved all variables considered for model 1 and model 2 (that is, 127 variables). Two-sided MWU was used for assessing the significance of group-wise comparisons using HC (*n* = 275) and ACS (*n* = 112) in MetaCardis population and HC (*n* = 473) versus ACS (*n* = 156) in the Israeli population. Multiple testing corrections were done using the Benjamini–Hochberg method, and FDR ≤ 0.1 was considered significant.

To validate our classification models further, we took advantage of the independent dataset from the companion paper^25^, focusing on our ACS subgroup to match the pathology of the Israeli study sample. ACS-specific metabolomics markers from the two studies were highly correlated (Cliff’s delta values computed relative to HC are shown in Fig. 6a and Supplementary Table 18), confirming that similar changes were observed in the two studies and validating a large fraction of our ACS-specific metabolome features. Notably, our markers exhibited strong discriminatory potential when employed in O-PLS-DA models trained in our population and tested in the independent Israeli population^25^. Models based on our ACS-specific metabolome markers with clinical variables (model 3, area under the receiver operating characteristic curve (AUROC) = 0.87) or without clinical variables (model 2, AUROC = 0.85) performed substantially better than a model based on clinical variables alone (AUROC = 0.764) (Fig. 6c). Altogether, our work confirmed the robustness of the discriminatory potential of our deconfounded microbiome and metabolome markers in a clinical setting (Fig. 6 (metabolome markers) and Extended Data Fig. 10 (microbiome and metabolome markers)).

## Discussion

We show that a vast majority of the intestinal microbiome and circulating and urine metabolome signatures that were previously reported as characteristic of IHD and that do not reduce to drug treatment effects are, in fact, already present in individuals with common dysmetabolic phenotypes, such as obesity and T2D. Our observations further align with the presence of a reduced gut bacterial cell density and changes in the abundance of multiple species and microbial functional potentials. Accounting for bacterial cell density, we identify the low-cell-count *Bacteroides 2* enterotype^23^ as a biomarker both in individuals with dysmetabolism and in individuals diagnosed with IHD. We particularly highlight low gut bacterial cell count as one of the microbiome features linked with IHD, which appears to reverse in treated IHD cases. Interestingly, both the present paper and another recent MetaCardis publication^42^ suggest that statin drugs widely prescribed to individuals with CMD might help restore gut bacterial cell load. These results are particularly relevant because several statins and their drug metabolites (mostly related to atorvastatin) and β-blockers (metroprolol and its metabolites) are reflected in the here-identified specific signatures of IHD and its subtypes.

In individuals with diagnosed IHD and treatment-induced improvement of vascular, inflammatory and lipid health markers, we found less aberrant microbiome and metabolome profiles when compared to healthy individuals. Still, we found bacterial species specifically altered in IHD cases, and most of them were depleted in agreement with findings of the companion paper^25^. Similarly, we observed a depletion of IHD-specific metabolites, including the fatty acid esters, ergothioneine and alpha-tocopherol, known for vasoprotective^31^ and antioxidant properties^32^, whereas metabolites enriched in individuals with IHD included intermediates related to TMA and compounds derived from tryptophan and phenylalanine metabolism. Finally, 4-cresol and phenylacetylglutamine stood out as representatives of ESCF, potentially mirroring disease severity.

In IHD subtype analyses, we identified multiple dysmetabolism-related gut microbiome changes in individuals with ACS, further strengthening our hypothesis that gut microbiome alterations take place in the prodromal stages before the onset of IHD. In contrast, a substantial fraction of altered host metabolites (45%) in individuals with ACS was unrelated to dysmetabolism. In addition, we found alterations of the microbiome and metabolome that were specific for CIHD and HF, putatively conditioned by a conjunction of intervention and disease worsening.

Of relevance for actionable targets in future preclinical and clinical trials, we confirm reduced microbiome potentials for biosynthesis of SCFAs and increased production of BCAAs^43^ in individuals at increased risk of asymptomatic coronary atherosclerosis before IHD diagnosis. In the later phases of IHD pathogenesis, we show an overwhelming role for microbial–host metabolism of aromatic amino acids derived from phenylalanine and tyrosine—that is, emerging from phenylacetate and cresol co-metabolism. Thus, our findings suggest that, beyond diminishing microbial–host production of TMAO, future interventions aiming to delay or prevent IHD might be directed at increasing microbial SCFA biosynthesis but lowering microbial production of aromatic amino acids and BCCAs. Finally, the identified microbiome and metabolome features allowed us to stratify individuals with IHD from healthy individuals or metabolically matched individuals at levels above those achieved with conventional risk markers, pointing to their pathophysiological relevance.

In conclusion, at prodromal dysmetabolic stages and at both early and late clinical manifestations of IHD, multiple deconfounded microbiome and metabolome alterations are present, reflecting distinct metabolic pathways. Several of these are modifiable and might be targets for future mechanistic experiments and clinical trials aiming at IHD prevention.

## Methods

### Study design and participants

The MetaCardis project included HCs and individuals at increasing stages of dysmetabolism and IHD severity, aged 18–75 years old and recruited from Denmark, France and Germany between 2013 and 2015. IHD cases were classified into patients with first case of ACS (<15 d); patients CIHD with normal heart function; and patients with documented HF and IHD as demonstrated by echocardiography-evaluated LVEF less than 45%. Our study encompassed 372 individuals with IHD (112 with ACS, 158 with CIHD and 102 with HF caused by CIHD). In addition, 275 HCs were matched on demographics, age and sex as well as 222 UMMCs—that is, individuals with features of the metabolic syndrome but receiving no lipid-lowering, anti-diabetic or anti-hypertensive drugs. Finally, we included 372 controls matched with individuals with IHD in terms of T2D status and BMI (referred to as MMCs).

Exclusion criteria were known confounders of the gut microbiome—that is, antibiotic use in the 3 months before inclusion, past history of abdominal cancer with and without radiation therapy, intestinal resection except for appendectomy and inflammatory or infectious diseases, including hepatitis B, hepatitis C or HIV.

Additionally, patients with a history of organ transplantation, patients receiving immunosuppressants, patients with estimated glomerular filtration rate (eGFR) <50 ml/min/1.73 m^2^ and patients with drug or alcohol addiction were excluded. Ethical approval was obtained from the Ethics Committee CPP Ile-de France, the Ethical Committees of the Capital Region of Denmark (H-3-2013-145) and the Ethics Committee at the Medical Faculty at the University of Leipzig (047-13-28012013). All study participants provided written informed consent, and all clinical investigations were undertaken according to Helsinki Declaration II.

### Bioclinical variables

Clinical measurements were made using standardized operating procedures concluded before patient recruitment. Bioclinical variables included age, sex, BMI, smoking status, dietary intake, physical activity and drug intake. We obtained habitual dietary information using food frequency questionnaires adapted to cultural habits of each of country of recruitment. Smoking status was obtained from a standardized questionnaire, and information on physical activity was assessed using the Recent Physical Activity Questionnaire. Drug intake was assessed either by recall or from medication list, and a medical specialist interviewed study participants about adherence to prescribed medication.

T2D was defined as fasting plasma glucose ≥7 mmol l^−1^ and/or Hba1c ≥6.5% and/or individuals taking any glucose-lowering agents. Hypertension was defined as systolic blood pressure >140 mmHg and/or diastolic blood pressure >90 mmHg and/or individuals taking anti-hypertensive drugs. Echocardiography enabled the measure of LVEF for diagnosis of HF (LVEF <45%). Renal function was assessed via eGFR calculated using the Modification of Diet in Renal Disease equation^44^.

Blood was collected in the morning after an overnight fast. Plasma and serum samples were stored at the clinical centers at −80°C until shipment to a central laboratory facility. Fasting plasma glucose, total and HDL cholesterol, triglycerides, creatinine and HbA1c levels were measured using standard enzymatic methods. LDL cholesterol concentrations were measured enzymatically for German participants or by the Friedwald equation for French and Danish participants. Alanine aminotransferase, aspartate aminotransferase and γ-glutamyltransferase were measured by enzyme-coupled kinetic assays. Ultra-sensitive C-reactive protein was measured using an Image Automatic Immunoassay System (Beckman Coulter). High-sensitivity IL-6 was measured using the Human IL-6 Quantikine HSELISA Kit (R&D Systems). IFN-γ-induced protein 10 (IP-10) and C-X-C motif chemokine ligand 5 (CXCL5), CCL2, Eotaxin, IL7, MIF, MIP1b, SDF1 and VEGFa were measured using a Luminex assay (ProcartaPlex Mix&Match Human 13-plex, eBioscience). Plasma pro-ANP was measured using a processing-independent assay^45^.

### Stool sample collection

Stool samples were processed according to International Human Microbiome Standards (IHMS) guidelines (SOP 03 V1). Samples were collected by study participants at home and immediately stored at −20 °C until they were transported on dry ice and frozen 4–24 h later at −80°C in plastic tubes at the biobanks of corresponding recruitment centers.

### Microbial load measurement by flow cytometry

Microbial loads of fecal samples were processed and analyzed as described^23^. In brief, 0.2-g frozen (−80 °C) aliquots were suspended in physiological solution to a total volume of 100 ml (8.5 g l^−1^ NaCl, VWR); the slurry was diluted 1,000 times; and samples were filtered using a sterile syringe filter (pore size 5 µm, Sartorius). Next, 1 ml of the microbial cell suspension was stained with 1 µl of SYBR Green I (1:100 dilution in DMSO, shaded 15-min incubation at 37 °C, 10,000 concentrate, Thermo Fisher Scientific). The flow cytometry analysis was performed using a C6 Accuri flow cytometer (BD Biosciences) based on Prest et al.^46^. Events were monitored using the FL1 533/30-nm and FL3 >670-nm detectors. Instrument and gating settings were kept identical for all samples (fixed staining/gating strategy^46^), and cell counts were converted to microbial loads per gram of fecal material (microbial load index).

### Stool sample processing and metagenomic analyses

Total fecal DNA was extracted following the IHMS guidelines (SOP 07 V2 H). Samples were sequenced in a non-randomized order using ion proton technology (Thermo Fisher Scientific) resulting in 23.3 ± 4.0 million (mean ± s.d.) single-end short reads with an on-average length of 150 bases. Sequencing was carried out with standardized protocols at a single site (Metagenopolis) over a period of 18 months. There was no significant bias of the sequencing date for different Metacardis groups (Kruskal–Wallis *P* value of 0.4 for HC, MMC, UMMS and IHD groups). Reads were cleaned using Alien Trimmer (version 0.4.0)^47^ to (1) remove resilient sequencing adapters and (2) trim low-quality nucleotides at the 3′ side using a quality and length cutoff of 20 bp and 45 bp, respectively. Cleaned reads were subsequently filtered from human and other possible food contaminant DNA using human genome RCh37-p10, *Bos taurus* and *Arabidopsis thaliana* with an identity score threshold of 97%. Gene abundance profiling was performed using the 9.9 million gene integrated reference catalog of the human microbiome^48^. Filtered high-quality reads were mapped with an identity threshold of 95% to the 9.9 million gene catalog using Bowtie2 (version 2.3.4)^49^ included in METEOR version 3.2 (https://forgemia.inra.fr/metagenopolis/meteor) software^50^. A gene abundance profiling table was generated by means of a two-step procedure using METEOR version 3.2. First, reads mapped to a unique gene in the catalog were attributed to their corresponding genes. Second, reads that mapped with the same alignment score to multiple genes in the catalog were attributed according to the ratio of their unique mapping counts. The gene abundance table was processed for rarefaction and normalization and further analysis using the MetaOMineR (momr, version 1.31) R package^5^. To decrease technical bias due to different sequencing depth and to avoid any artifacts of sample size on low-abundance genes, read counts were rarefied. The gene abundance table was rarefied to 10 million reads per sample by random sampling of 10 million mapped reads without replacement. The resulting rarefied gene abundance table was normalized according to the fragments per kilobase of transcript per million mapped reads (FPKM) approach to give the gene abundance profile table.

Metagenomic species (MGS) are co-abundant gene groups with more than 500 genes corresponding to microbial species. In total, 1,436 MGS were clustered from 1,267 human gut microbiome samples used to construct the 9.9 million gene catalog^48^. MGS abundances were estimated as the mean abundance of the 50 genes defining a robust centroid of the cluster (if more than 10% of these genes gave positive signals). Abundances were corrected for bacterial cell count by multiplying by an index factor calculated as the bacterial cell count of the sample divided by the mean value of this bacterial cell count over the dataset as a whole. MGS taxonomical annotation was performed using all genes by sequence similarity using NCBI blast N; a species-level assignment was given if more than 50% of the genes matched the same reference genome of the NCBI database (November 2016 version) at a threshold of 95% of identity and 90% of gene length coverage. Remaining MGS were assigned to a given taxonomical level from genus to super-kingdom level, if more than 50% of their genes had the same level of assignment. MGS richness (MGS count) was calculated directly from the rarefied MGS abundance matrix. Bacterial gene richness (gene count) was calculated by counting the number of genes detected at least once in a given sample, using the average number of genes counted in ten independent rarefaction experiments. MGS richness (MGS count) was calculated directly from the rarefied MGS abundance matrix.

### Customized microbial module analysis

Customized module sets included previously described GMMs^51^ covering bacterial and archaeal metabolism specific to the human gut environment with a focus on anaerobic fermentation processes, expanded with a specific set of six modules zooming in on bacterial TMA metabolism^52^. Additionally, after a previously published strategy to build manually curated gut-specific metabolic modules^51,53^, we constructed a novel set of 20 modules describing microbial phenylpropanoid metabolism (phenylpropanoid metabolism modules) from shotgun metagenomic data. Abundances of customized modules were derived from the ortholog abundance tables using Omixer-RPM version 1.0 (https://github.com/raeslab/omixer-rpm)^51,54^. The coverage of each metabolic variant encoded in a module was calculated as the number of steps for which at least one of the orthologous groups was found in a metagenome, divided by the total number of steps constituting the variant. The coverage of the GMM was defined as equal to the one of the variants with maximum coverage. Module presence/absence was identified with a detection threshold of more than 66% coverage to provide tolerance to mis-annotations and missing data in metagenomes. Module abundance was calculated as the median of KEGG orthology abundance in the pathway with maximum coverage. Abundances were corrected for bacterial cell count similarly to MGSs.

### Metabolic profiling

We deployed a comprehensive metabolic phenotyping strategy combining in-house analysis by proton nuclear magnetic resonance (^1^H-NMR) spectroscopy, gas chromatography coupled to mass spectrometry (GC–MS) and targeted UPLC–MS/MS with untargeted UPLC–MS data generated by Metabolon, as described in detail below:

#### ^1^H NMR spectroscopy

^1^H-NMR experiments were carried out using a Bruker Avance spectrometer operating at 600 MHz, as reported^55–57^. Structural assignment was performed using data from literature, the Human Metabolome Database (http://www.hmdb.ca/), S-Base (Bruker) and in-house databases^55^. ^1^H-NMR spectra were pre-processed and exported to MATLAB for multivariate statistical analyses using O-PLS-DA, as previously reported^58^. Absolute metabolite quantifications were also derived using Bruker’s In Vitro Diagnostics for research (IVDr) quantification algorithms.

#### GC–MS semi-targeted profiling

Serum samples were prepared, analyzed and processed using standard protocols. In brief, serum samples (100 μl) were cleaned up with methanol protein precipitation, evaporated to dryness, derivatized and injected to an Agilent 7890B-5977B Inert Plus GC–MS system. The chromatographic column was an Agilent ZORBAX DB5-MS (30 m × 250 µm × 0.25 µm + 10 m Duragard). The temperature gradient was 37.5 min long, and the mass analyzer was operated in full-scan mode between 50 *m*/*z* and 600 *m*/*z*. Peaks were annotated with the use of the Fiehn library (Agilent G1676AA Fiehn GC/MS Metabolomics RTL Library, User Guide, Agilent Technologies, https://www.agilent.com/cs/library/usermanuals/Public/G1676-90001_Fiehn.pdf). Metabolic features with high reproducibility (CV <30%) and linearity (that is, dilution signal rho > 0.9 and false discovery rate (FDR)-corrected *P* < 0.05 (one-tailed Spearman)) were kept in the final dataset, resulting in 102 annotated metabolic features.

#### UPLC–MS/MS isotopic quantification of methylamines and carnitines

UPLC–MS/MS was employed for the determination of absolute concentrations for TMA, TMAO, choline, betaine, γ-butyrobetaine, betaine-aldehyde, butyryl-carnitine, isovaleryl-carnitine, OH-isovaleryl-carnitine, stearoyl-carnitine, oleoyl-carnitine, linoleoyl-carnitine, myristoyl-carnitine, lauroyl-carnitine and decanoyl-carnitine.

Serum samples (50 μl) were prepared as follows: (1) samples were spiked with 10 μl of Internal Standard solution (^13^C_3_/^15^N-TMA, d_9_-TMAO, d_4_-choline, d_9_-isovaleryl carnitine and d_9_-betaine in water, 1 mg l^−1^; Sigma-Aldrich); (2) 30 μl of ethyl-2-bromoacetate solution (22.5 g l^−1^ of ethyl-2-bromoacetate and 1.4% NH_4_OH in acetonitrile) was added, and derivatization of trimethylamines (TMA and ^13^C_3_/^15^N-TMA) to their ethoxy-analogues was completed after 30 min at room temperature; and (3) 910 μl of protein/lipid precipitation solution (94% acetonitrile/5% water/1% formic acid) was added; samples were centrifuged for 20 min (4 ^°^C at 20,000*g*); and 400 μl of the supernatants was transferred to UPLC autosampler 500-μl-well plates. Sample injections (5 μl, full loop) were performed to a Waters Acquity UPLC-Xevo TQ-S UPLC–MS/MS system equipped with an Acquity BEH HILIC (2.1 × 100 mm, 1.7 µm) chromatographic column. An isocratic elution was applied with 10 mM ammonium formate in 95:5 (v/v) acetronitrile:water for 11.5 min at 500 μl ml^−1^ and 50 ^°^C. Positive electrospray (ESI^+^) was used as ionization source, and mass spectrometer parameters were set as follows: capillary, cone and sources voltages at −700 V, −18 V and 50 V, respectively; desolvation temperature at 600 ^°^C; and desolvation/cone/nebulizer gases were high-purity nitrogen at 1,000 L h^−1^, 150 L h^−1^ and 7 bar, respectively. Collision gas was high-purity argon. Mass spectrometer was operated in multiple reaction monitoring mode. The monitored transitions were the following: for derivatized TMA, +146>+118/59 *m*/*z* (23/27 V); for derivatized ^13^C_3_/^15^N-TMA, +150>+63/122 *m*/*z* (27/22 V); for TMAO, +76>+59/58 *m*/*z* (12/13 V); for d_9_-TMAO, +85>+68/66 *m*/*z* (18/20 V); for choline, +104>+60/45 *m*/*z* (20/22 V); for d_4_-choline, +108>+60/45 *m*/*z* (20/22 V); for isovaleryl-carnitine, +246>+85/145 *m*/*z* (19/19 V); for d_9_-isovaleryl-carnitine, +255>+85 *m*/*z* (19 V); for betaine, +118>+59/73 *m*/*z* (18/19 V); for d_9_-betaine, +127>+68 *m*/*z* (19 V); for γ-butyrobetaine, +146>+87/60 *m*/*z* (17/19 V); for betaine-aldehyde, +103>+60.5/74 *m*/*z* (12/12 V); for butyryl-carnitine, +232>+85/173 *m*/*z* (14/12 V); for OH-isovaleryl-carnitine, +262>+86/61 *m*/*z* (20/20 V); for stearoyl-carnitine, +428>+86/371 *m*/*z* (21/17 V); for oleoyl-carnitine, +426>+86/61 *m*/*z* (22/22 V); for linoleoyl-carnitine, +424>+86/69 *m*/*z* (24/24 V); for myristoyl-carnitine, +372.5>+86/61 *m*/*z* (24/24 V); for lauroyl-carnitine, +344.5>+86/61 *m*/*z* (21/21 V); and for decanoyl-carnitine, +316.5>+86/145 *m*/*z* (21/21 V). The system was controlled by MassLynx (version 4.2, Waters) software, also used for the data acquisition and analysis.

#### UPLC–MS untargeted profiling

Serum samples were extracted and profiled by Metabolon using a UPLC–MS-based methodology^59^. Annotated metabolites and unknown features (denoted X-00000) were identified by comparing sample features with ion features in a reference database of pure chemical standards and previously detected unknowns, followed by detailed visual inspection and quality control, as reported^60^.

For all metabolomic assays, we randomized the sample preparation order across the whole study so that each sample preparation batch included samples from all study groups. For MS untargeted assays, median batch correction was performed by adjusting batch-wise study sample variable medians according to a scalar derived from adjusting pooled reference sample medians, so that pooled reference sample medians are identical across all batches.

The randomized sample preparation batches were also tested for association with study groups using univariate statistics (Fisher’s exact test or Kruskal–Wallis test), and *P* > 0.05 was observed across all methods (GC–MS, Fisher’s exact test, *P* = 0.23; UPLC–MS targeted, Fisher’s exact test, *P* = 0.12; and UPLC–MS untargeted (Metabolon), Fisher’s exact test, *P* = 0.65). In addition, NMR run order exhibited a Kruskal–Wallis *P* = 0.49. To choose a single measurement for the duplicate metabolites observed across platforms, we prioritized measurements based on the analytical quality of the data as follows:

1. Targeted quantification using isotopic standards (for example, UPLC–MS/MS for acylcarnitines and TMA)
2. Relative abundance with structural ID confirmed by native standards (for example, Metabolon UPLC–MS)
3. Relative/absolute or quantification by NMR calibrated against a database/reference dataset (for example, > NMR quantifications and manually assigned peaks)
4. Relative or quantification with metabolite ID check against a standards database (for example, GC–MS)

### Drug deconfounding analysis

The pipeline was used to assess to what extent observed differences among groups of study participants in microbiome, metabolome and bioclinical feature abundance are confounded, in the sense of being consequences of other (treatment or risk factor) variables different among the groups more so than characteristic of the specific phenotype itself. We employed the post hoc filtering approach implemented in the R package metadeconfoundR (version 0.1.8; see https://github.com/TillBirkner/metadeconfoundR or 10.5281/zenodo.4721078) that was devised within the MetaCardis consortium^8^.

The pipeline has two steps. In the first, all associations between -omics features and the set of independent variables (disease status, drug treatment status and risk markers, including age and smoking status) are determined under non-parametric statistics (Mann–Whitney *U*-test (MWU) or Spearman test, adjusted for multiple testing using the Benjamini–Hochberg method). For each feature significantly (FDR < 0.1) associated with defined phenotype status, it is checked whether it has significant associations with any potential confounder. If not, it is considered trivially unconfounded (not confounded (NC)). If at least one covariate also has significant association with the feature, then, for each such covariate, a post hoc test for confounding is applied. This test takes the form of nested linear model comparisons (likelihood ratio test for *P* values), where the dependent variable is the feature (X), and the independent variables are the disease status (A) and the tested covariate (B) versus a model containing only the covariate (B), thus testing whether disease status (A) adds explanatory statistical power beyond the covariate (B). If this holds (likelihood ratio test (LRT) *P* < 0.05) for all covariates (B), then disease status is confidentially deconfounded (CD) concerning its effect on feature X; it cannot be reduced to any confounding factor. For each covariate (B) where significance is lost, a complementary modeling test is performed of the complementary model pairs, predicting (X) as a function of (A) and (B) versus a model containing (A) alone, thus testing whether the covariate (B) in turn is equally reducible to (A). If for at least one such covariate (B), (B) has independent effect (LRT *P* < 0.05) on top of (A), then the feature (X) is considered confounded by (B). However, if in none of the pairwise tests the original significance holds, then (A) and (B) are considered so correlated that their relative influence cannot be disentangled. We consider these cases laxly deconfounded (LD), in the sense that, for these cases, clear confounding influence can neither be concluded nor ruled out. The R package was applied to the present dataset considering medication status either as binary variables or as normalized dosages.

Our deconfounding pipeline takes into account linear effects related to drug categories. Still, we were not able to control for every possibly lifestyle confounding factor, making a lack of full confounding adjustment a limitation of our study.

### Statistical analyses

Downsampled microbiome functional profile and taxonomic composition data, metabolite and quantitative clinical phenotype measurements were assessed between and within groups using non-parametric tests (MWU and Spearman test) corrected for multiple testing using the Benjamini–Hochberg approach. All tests undertaken as part of the univariate biomarker analyses involved comparing only two groups. The main exception was the comparison between the three study centers where we applied a Kruskal–Wallis test. Non-parametric directional standardized effect sizes were likewise taken as the Cliff’s delta and Spearman rho, respectively. Classification models were built using multivariate O-PLS-DA using the ropls R package. ROC analysis was performed using the ROCR package. To control for influence of covariates associated with disease severity, including sex, smoking, dietary indices and drug treatment, a post hoc test approach was adopted as outlined above. R packages, including lmtest, orddom, ropls, ROCR, circlize, ggplot2, PCMCR using R version 4.0.2 and RStudio versions 1.4.1717 and 1.2.5033, were used for various analyses.

### Reporting Summary

Further information on research design is available in the Nature Research Reporting Summary linked to this article.

## Online content

Any methods, additional references, Nature Research reporting summaries, source data, extended data, supplementary information, acknowledgements, peer review information; details of author contributions and competing interests; and statements of data and code availability are available at 10.1038/s41591-022-01688-4.

## Supplementary information

Supplementary Information Supplementary Methods and Supplementary Figs. 1 and 2.

Reporting Summary

Supplementary Tables 1–18 Legends and details of 18 Supplementary tables.

## Data Availability

Supplementary information on data availability is linked to the online version of the paper at www.nature.com/nature. Raw shotgun sequencing data that support the findings of this study have been deposited in the European Nucleotide Archive with accession codes PRJEB37249, PRJEB38742, PRJEB41311 and PRJEB46098, with public access. Metabolome data have been uploaded to Metabolights and MassIVE with respective accession numbers—that is, serum NMR and urine NMR with accession number MTBLS3429, serum GCMS with accession number MassIVE MSV000088042 and additional isotopically quantified serum metabolites using UPLC–MS/MS with accession number MassIVE MSV000088043. Processed pseudonymized per-subject -omics and metadata are provided in Supplementary Tables 9–13, and medication profiles are given in Supplementary Table 14.

## Code availability

The novel drug-aware univariate biomarker testing pipeline, described in full elsewhere^8^, is available as an R package (metadeconfoundR; Birkner et al., manuscript in preparation) on GitHub (https://github.com/TillBirkner/metadeconfoundR) and also at 10.5281/zenodo.4721078. The latest version (0.1.8) of this package was used to generate the data shown in this publication. In addition, the scripts using this package to perform the analysis presented here are available at 10.5281/zenodo.5516219.
