# Supplementary material for: Microbiome and metabolome features of the cardiometabolic disease spectrum
Source: Nat Med. 2022 Feb 17;28(2):303–14. doi: 10.1038/s41591-022-01688-4 (PMC8863577; doi:10.1038/s41591-022-01688-4)
Supplement: Supplementary file 1 — Supplementary Methods and Supplementary Figs. 1 and 2. [file 41591_2022_1688_MOESM1_ESM.pdf]

nature portfolio

https://doi.org/10.1038/s41591-022-01688-4

Supplementary information

Microbiome and metabolome features of the cardiometabolic disease spectrum

In the format provided by the authors and unedited

nature portfolio

https://doi.org/10.1038/s41591-022-01688-4

Supplementary information

Microbiome and metabolome features of the cardiometabolic disease spectrum

In the format provided by the authors and unedited

Supplementary Material

Microbiome and Metabolome Features of the Cardiometabolic Disease Spectrum

Authors:

Sebastien Fromentin[1,*], Sofia K. Forslund[2,3,4,5,6,*], Kanta Chechi[7,8,34*], Judith Aron-Wisnewsky[9,10,*], Rima Chakaroun[11,*], Trine Nielsen[12,*], Valentina Tremaroli[13], Boyang Ji[14], Edi Prifti[15,16], Antonis Myridakis[7], Julien Chilloux[7], Andrikopoulos Petros[7,34], Yong Fan[12] , Michael T. Olanipekun[7], Renato Alves[2], Solia Adiouch[9], Noam Bar[18,19], Yeela Talmor-Barkan[18,19,20,21], Eugeni Belda[9,15,16], Robert Caesar[13], Luis Pedro Coelho[2], Gwen Falony[22,23], Soraya Fellahi[24], Pilar Galan[1], Nathalie Galleron[1], Gerard Helft[25], Lesley Hoyles[7,26], Richard Isnard[25], Emmanuelle Le Chatelier[1], Hanna Julienne[1], Lisa Olsson[13], Helle Krogh Pedersen[12], Nicolas Pons[1], Benoit Quinquis[1], Christine Rouault[9], Hugo Roume[1], Joe-Elie Salem[27], Thomas SB Schmidt[2], Sara Vieira-Silva[22,23], Peishun Li[14], Maria Zimmermann-Kogadeeva[2], Christian Lewinter[28], Nadja B Søndertoft[12], Tue H Hansen[12], Dominique Gauguier[29], Jens Peter Gøtze[30], Lars Køber[28], Ran Kornowski[20], Henrik Vestergaard[12,31], Torben Hansen[12], Jean-Daniel Zucker[15,17], Serge Hercberg[32], Ivica Letunic[33], Fredrik Bäckhed[12,13], Jean-Michel Oppert[10], Jens Nielsen[14], Jeroen Raes[22,23], Peer Bork[2], Michael Stumvoll[11], Eran Segal[18,19], Karine Clément[9,10,+], Marc-Emmanuel Dumas[7,34,35,+], S. Dusko Ehrlich[1,+], and Oluf Pedersen[12,+]

Collaborators in the MetaCardis Consortium:

Karen Asmann, Chloe Amouyal, Ehm Astrid Andersson Galijatovic, Fabrizio Andreelli, Olivier Barthelemy, Jean-Philippe Bastard, Jean-Paul Batisse, Magalie Berland, Randa Bittar, Hervé Blottière, Frederic Bosquet, Rachid Boubrit, Olivier Bourron, Mickael Camus, Dominique Cassuto, Cecile Ciangura, Jean-Philippe Collet, Maria-Carlota Dao, Morad Djebbar, Angélique Doré, Line Engelbrechtsen, Leopold Fezeu, Philippe Giral. Agnes Hartemann, Bolette Hartmann, Malene Hornbak, Jean-Sebastien Hulot, Sophie Jaqueminet, Bridget Holmes, Jens J. Holst, Niklas

Rye Jørgensen, Johanne Justesen, Judith Kammer, Nikolaj Karup, Mathieu Kerneis, Jean Khemis, Ruby Kozlowski, Véronique Lejard, Florence Levenez, Lea Lucas-Martini, Robin Massey, Laura Martinez-Gili, Nicolas Maziers, Jonathan Medina-Stamminger, Gilles Montalescot, Sandrine Moutel, Ana Luisa Neves, Hoai Tuong Nguyen, Laetitia Pasero Le Pavin, Christine Poitou, Francoise Pousset, Laurence Pouzoulet, Andrea Rodriguez-Martinez, Johanne Silvain, Mathilde Svendstrup, Timothy Swartz, Thierry Vanduyvenboden, Camille Vatier, Eric Verger, Stefanie Walther.

Contents

Supplementary Methods

Supplementary Figure 1: Distribution of metagenomic and metabolomic features among HC, MMC and IHD groups as per the classification scheme

Supplementary Figure 2: Distribution of metagenomic and metabolomic features among HC, MMC and IHD sub-groups as per the classification scheme

Supplementary Methods

Categorization of microbiome and metabolome features across the CMD spectrum:

The deconfounded features were systematically evaluated (see **Extended Data Figure 4**) based on statistical significance, similarity of effect size, and directional alignment of microbiome and metabolome features in the various group comparisons revealing dysmetabolic-, escalation-, de-escalation- and IHD-specific features as well as subgroup-specific features (also see Figure 3 for visualization of the approach). The categorization was achieved as following:

Root: Test HC vs IHD:

1. If HC vs IHD is significant, also test HC vs MMC:

    1.1. If HC vs MMC is not significant, test MMC vs IHD:

        1.1.1. If MMC vs IHD is significant: IHD-specific marker.

        1.1.2. If MMC vs IHD is not significant:

        1.1.2.1. If both HC vs UMMC and UMMC vs IHD are significant and HC vs

        IHD is congruent with HC vs MMC: IHD-escalation marker

        1.1.2.2. If both HC vs UMMC and UMMC vs IHD are significant and HC vs IHD is incongruent with HC vs MMC: IHD de-escalation marker

        1.1.2.3. If HC vs UMMC is non-significant but UMMC vs IHD is significant:

        IHD-specific marker.

        1.1.2.4. If HC vs UMMC is significant but UMMC vs IHD is insignificant:

        dysmetabolism marker.

        1.1.2.5. HC vs UMMC and UMMC vs IHD are insignificant: IHD-specific marker

    1.2. If HC vs MMC is significant, test MMC vs IHD:

        1.2.1. If all three are significant, and HC vs MMC is congruent with MMC vs IHD: IHD escalation marker.

1.2.2. If all three are significant, but HC vs MMC is incongruent with MMC vs
IHD: IHD de-escalation marker.

1.2.3. If HC vs IHD, HC vs MMC are significant but not MMC vs IHD, and HC vs MMC is congruent with HC vs IHD: dysmetabolism marker

1.2.4. If HC vs IHD, HC vs MMC are significant but not MMC vs IHD, and HC vs MMC is incongruent with HC vs IHD: IHD de-escalation marker

2. If HC vs IHD is not significant but MMC vs IHD is significant, test HC vs MMC

2.1. If HC vs MMC is significant, compare directions of HC vs MMC and MMC vs IHD.

2.1.1. If HC vs MMC is congruent with MMC vs IHD: IHD escalation marker

2.1.2. If HC vs MMC is incongruent with MMC vs IHD: IHD de-escalation marker

2.2. If HC vs MMC is not significant, test HC vs UMMC and UMMC vs IHD

2.2.1. If both HC vs UMMC and UMMC vs IHD are significant and MMC vs
IHD is congruent with HC vs MMC: IHD escalation marker

2.2.2. If both HC vs UMMC and UMMC and IHD are significant and MMC vs
IHD is incongruent with HC vs MMC: IHD de-escalation marker

2.2.3. If HC vs UMMC is not significant but UMMC vs IHD is significant: IHDspecific marker

2.2.4. If HC vs UMMC is significant but UMMC vs IHD is not significant and
MMC vs IHD is congruent with HC vs MMC: IHD escalation marker

2.2.5. If HC vs UMMC is significant but UMMC vs IHD is not significant and MMC vs IHD is incongruent with HC vs MMC: IHD de-escalation marker.

2.2.6. If both HC vs UMMC and UMMC vs IHD are insignificant: IHD-specific marker.

3. If HC vs IHD is NS, MMC vs IHD is NS but only HC vs MMC is significant: metabolic dysregulation marker.

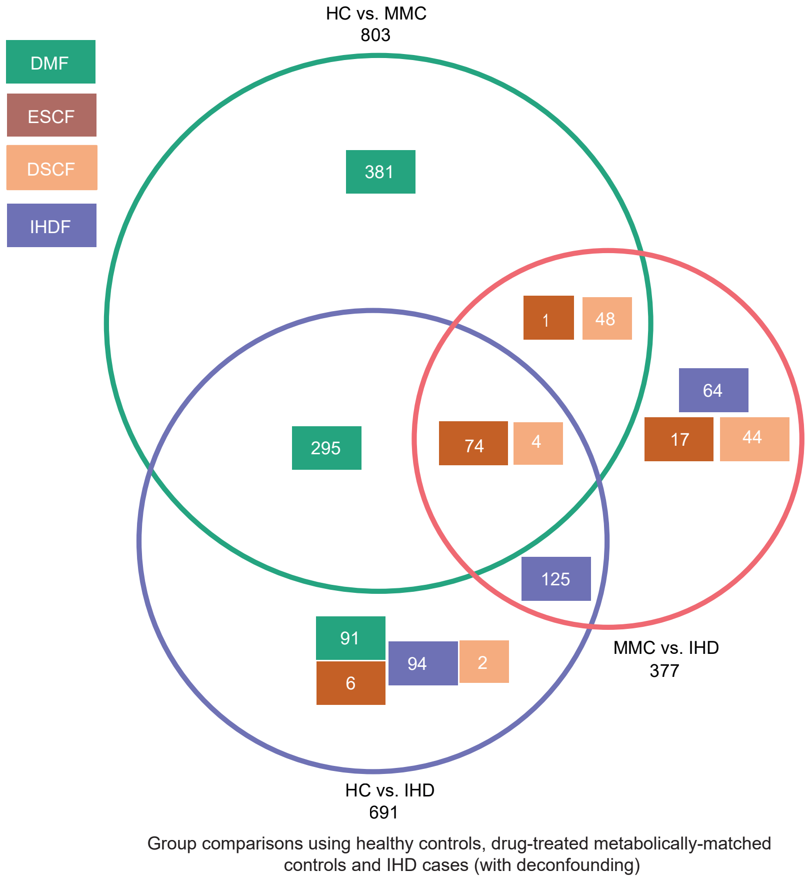

Supplementary Figure 1: Distribution of metagenomic and metabolomic features among HC, MMC and IHD groups as per the classification scheme (also covered in Supplementary Table 17). Venn diagram showing the distribution of IHDF, DMF, ESCF and DSCF derived from the application of classification scheme (Extended Data Figure 4) to the HC (n = 275), drug-treated MMC (n = 372) and IHD cases (n = 372). IHD: ischemic heart disease patients, HC: healthy controls, MMC: metabolically matched controls, IHDF: IHD specific features, ESCF: escalation features, DSCF: De-escalation features, DMF: dysmetabolism features.

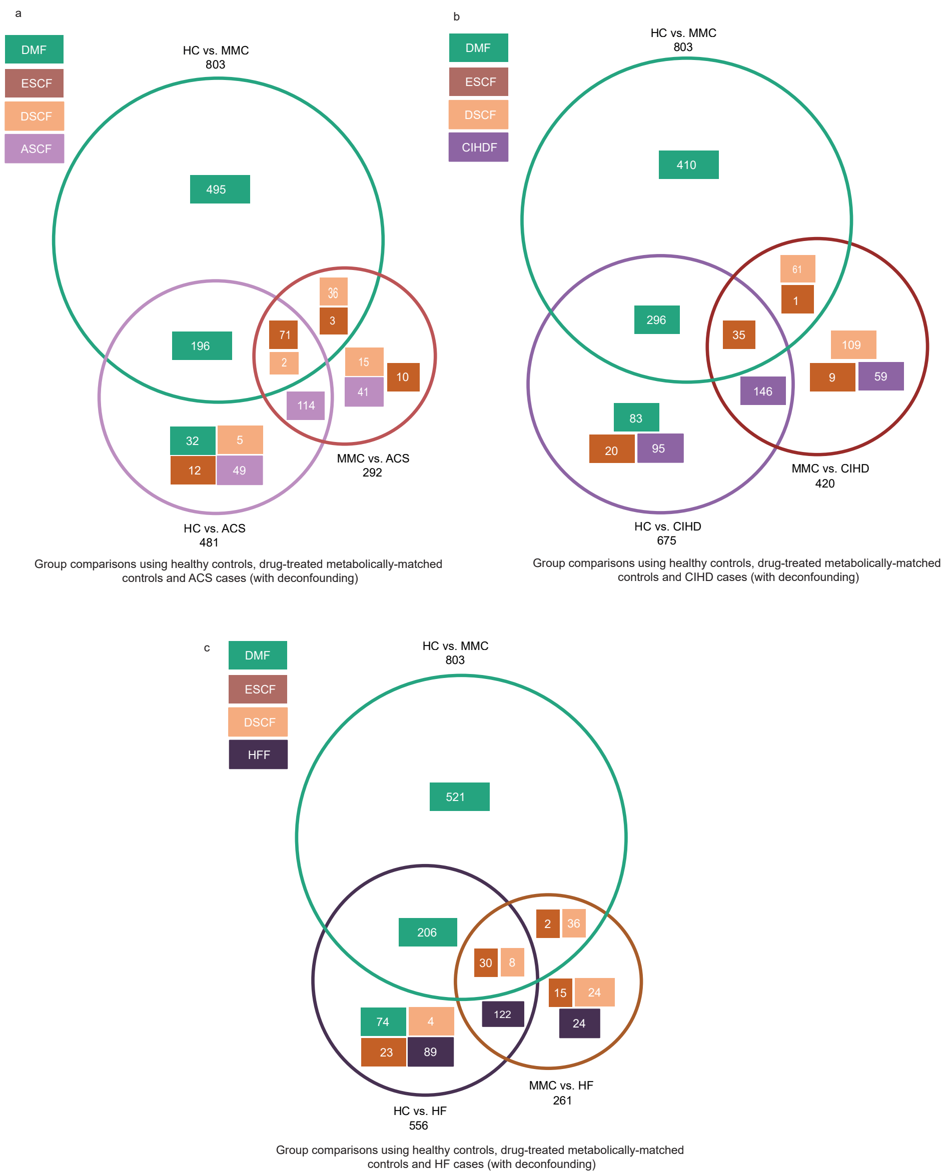

Supplementary Figure 2: Distribution of metagenomic and metabolomic features among HC, MMC and IHD sub-groups as per the classification scheme (also covered in Supplementary Table 17). Venn diagram showing the distribution of subgroup specific, ESCF, DSCF and DMF when classification scheme (Extended Data Figure 4) was applied to ACS (n = 112), CIHD (n = 158) and HF (n = 102) groups relative to drug-treated MMC (n = 372) and HC (n = 275). HC: healthy controls, MMC: metabolically matched controls, ACS: acute coronary syndrome, CIHD: chronic IHD, HF: heart failure due to CIHD, ACSF: ACS specific features, CIHDF: CIHD specific features, HF specific features, ESCF: escalation features, DSCF: De-escalation features, DMF: dysmetabolism features.
