# Supplementary material for: Microbiome and metabolome features of the cardiometabolic disease spectrum
Source: Nat Med. 2022 Feb 17;28(2):303–14. doi: 10.1038/s41591-022-01688-4 (PMC8863577; doi:10.1038/s41591-022-01688-4)
Supplement: Supplementary file 2 — Reporting Summary [file 41591_2022_1688_MOESM2_ESM.pdf]

nature research

Corresponding author(s): Oluf Pedersen
S. Dusko Ehrlich
Karine Clément
Marc-Emmanuel Dumas

Last updated by author(s): Dec 26, 2021

Reporting Summary

Nature Research wishes to improve the reproducibility of the work that we publish. This form provides structure for consistency and transparency in reporting. For further information on Nature Research policies, see our Editorial Policies and the Editorial Policy Checklist.

Statistics

For all statistical analyses, confirm that the following items are present in the figure legend, table legend, main text, or Methods section.

n/a	Confirmed	
☐	☒	The exact sample size (*n*) for each experimental group/condition, given as a discrete number and unit of measurement
☒	☐	A statement on whether measurements were taken from distinct samples or whether the same sample was measured repeatedly
☐	☒	The statistical test(s) used AND whether they are one- or two-sided<br>*Only common tests should be described solely by name; describe more complex techniques in the Methods section.*
☐	☒	A description of all covariates tested
☐	☒	A description of any assumptions or corrections, such as tests of normality and adjustment for multiple comparisons
☐	☒	A full description of the statistical parameters including central tendency (e.g. means) or other basic estimates (e.g. regression coefficient) AND variation (e.g. standard deviation) or associated estimates of uncertainty (e.g. confidence intervals)
☐	☒	For null hypothesis testing, the test statistic (e.g. *F*, *t*, *r*) with confidence intervals, effect sizes, degrees of freedom and *P* value noted<br>*Give P values as exact values whenever suitable.*
☒	☐	For Bayesian analysis, information on the choice of priors and Markov chain Monte Carlo settings
☒	☐	For hierarchical and complex designs, identification of the appropriate level for tests and full reporting of outcomes
☐	☒	Estimates of effect sizes (e.g. Cohen's *d*, Pearson's *r*), indicating how they were calculated

Our web collection on statistics for biologists contains articles on many of the points above.

Software and code

Policy information about availability of computer code

Data collection	See MS and Online Methods for details; METEOR v3.2 (https://forgemia.inra.fr/metagenopolis/meteor), Alientrimmer:v0.4.0, Bowtie2 v2.3.4, MetaOMineR (momr, v1.31), Omixer-RPM (v1.0) were each used to process microbiome data. MassLynxTM (Waters corporation; Version 4.2) software was used for UPLC-MS/MS data acquisition and analysis.
Data analysis	Most analysis was conducted using the R statistical language as described in Methods and Online Methods. In particular the package metadeconfoundR (v0.1.8 - see https://github.com/TillBirkner/metadeconfoundR or https://doi.org/10.5281/zenodo.4721078) was employed. In addition we applied custom R and Perl scripts (see Code Availability or https://doi.org/10.5281/zenodo.5516219).

For manuscripts utilizing custom algorithms or software that are central to the research but not yet described in published literature, software must be made available to editors and reviewers. We strongly encourage code deposition in a community repository (e.g. GitHub). See the Nature Research guidelines for submitting code & software for further information.

Data

Policy information about availability of data

All manuscripts must include a data availability statement. This statement should provide the following information, where applicable:

- Accession codes, unique identifiers, or web links for publicly available datasets
- A list of figures that have associated raw data
- A description of any restrictions on data availability

Supplementary Information on data availability is linked to the online version of the paper at www.nature.com/nature. Raw shotgun sequencing data that support the findings of this study have been deposited in European Nucleotide Archive with accession codes PRJEB37249, PRJEB38742, PRJEB41311, and PRJEB46098 with public access. Metabolome data have been uploaded to Metabolights and MassIVE with respective accession numbers i.e., serum UPLCMS, serum NMR and urine NMR with accession number MTBLS3429, serum GCMS with accession number MassIVE MSV000088042, and additional isotopically quantified serum metabolites

using UPLC-MS/MS with accession number MassIVE MSV000088043. Processed pseudonymized per-subject -omics and metadata are provided in Supplementary Tables 9-13, and medication profiles are given in Supplementary Table 14.

Field-specific reporting

Please select the one below that is the best fit for your research. If you are not sure, read the appropriate sections before making your selection.

☒ Life sciences ☐ Behavioural & social sciences ☐ Ecological, evolutionary & environmental sciences

For a reference copy of the document with all sections, see nature.com/documents/nr-reporting-summary-flat.pdf

Life sciences study design

All studies must disclose on these points even when the disclosure is negative.

Sample size	No prior power calculation was carried out but sample size was selected so as to exceed that of the MetaHIT study, which was adequately powered.
Data exclusions	No subjects for which data was available was excluded during analysis.
Replication	The study was hypothesis generating and observational, not experimental. Reproducibility of findings was assessed by comparing present findings with 1) prior literature and 2) another ACS cohort from the back to back Talmor-Bar et al. paper 3) assessment of whether each association is reproduced (significant or trending) in the different disease subcohorts. Where testable, most findings replicate in these assessments. In more detail, as we report several hundred findings, the subsequent reproduction/consistency status of each is listed in the manuscript itself as well as in Supplementary Table 15, 16 and 18, respectively.
Randomization	As no intervention or experiment was made, only observation, there is no intervention to randomize and as such randomization is neither well-defined, applicable, meaningful or relevant.
Blinding	Investigators (clinicians and study nurses) were aware of clinical diagnosis by necessity and default, but blinded to any laboratory, clinical or -omics data as that was generated by others from biosamples. Analysts (data managers, statisticians, bioinformaticicans) were blinded by having access only to pseudonymized data, and performed no manual analyses - all statistics and visualization were undertaken using computer software not taking any group allocation into account except where testing for associations to it. As such, no analysts awareness of any group allocation (diagnosis) affected outcomes of any statistical analysis, and only results of such analysis in aggregate were used to draw conclusions and for interpretations of results. In this sense analysis is as blinded as is at all possible in an -omics biomarker study, and in line with standards of the field.

Reporting for specific materials, systems and methods

We require information from authors about some types of materials, experimental systems and methods used in many studies. Here, indicate whether each material, system or method listed is relevant to your study. If you are not sure if a list item applies to your research, read the appropriate section before selecting a response.

Materials & experimental systems

n/a	Involved in the study	
☒	☐	Antibodies
☒	☐	Eukaryotic cell lines
☒	☐	Palaeontology and archaeology
☒	☐	Animals and other organisms
☐	☒	Human research participants
☐	☒	Clinical data
☒	☐	Dual use research of concern

Methods

n/a	Involved in the study	
☒	☐	ChIP-seq
☒	☐	Flow cytometry
☒	☐	MRI-based neuroimaging

Human research participants

Policy information about studies involving human research participants

Population characteristics	The European MetaCardis project included healthy control individuals and individuals at increasing stages of dysmetabolism and ischaemic heart disease (IHD) severity, aged 18–75 years old, and recruited from Denmark, France and Germany between 2013 and 2015. IHD cases were recruited solely in Denmark and France and were classified into: patients with first case of acute coronary syndrome (<15 days) (ACS), patients with chronic IHD but normal heart function and another similar group with documented heart failure (HF) and IHD as demonstrated by left ventricular ejection fraction (LVEF) < 45% evaluated with echocardiography. In total, the study encompassed 372 IHD cases including 112 with acute coronary syndrome (ACS), 158 with chronic ischaemic heart disease (CIHD) and 102 with combined ischaemic heart disease and heart failure (HF). In addition, we included 275 healthy controls (HC) matched on demographics, age and sex, and 222 untreated metabolically matched controls (UMMC); i.e. individuals with features of the metabolic syndrome and thus at increased risk

	of IHD but receiving no lipid-lowering or anti-diabetic or anti-hypertensive drugs. Finally, we included 372 controls matched with IHD cases on T2D status and body mass index (BMI), thereafter termed metabolically matched controls (MMC). A large number of covariate-relevant population characteristics was tracked. These are described in Supplementary Tables 1-4.
Recruitment	The applied recruitment scheme resulted in a proband/patient population that match individuals who do/do not require care for the diseases in question, which does not credibly introduce any biases with bearing on the specific questions the study asks. While there is an uneven distribution of individuals from different clinical groups between the study sites, study site (France, Germany, Denmark) was included as a covariate, with findings reducible to the influence of this variable filtered out. As such, the recruitment strategy is not likely to bias the results.
Ethics oversight	The study protocol was approved by the Ethics Committee at the Medical Faculty at the University of Leipzig, the Ethical Committee of the Capital Region of Denmark and the Ethics Committee CPP Ile-de France. All participants provided written informed consent and all investigations were conducted according to Helsinki declaration..

Note that full information on the approval of the study protocol must also be provided in the manuscript.

Clinical data

Policy information about clinical studies

All manuscripts should comply with the ICMJE guidelines for publication of clinical research and a completed CONSORT checklist must be included with all submissions.

Clinical trial registration	The study protocol was registered at clinicaltrial.gov (NCT02059538).
Study protocol	Available from the study promoter: Assistance Publique-Hôpitaux de Paris (AP-HP).
Data collection	This is described in greater detail in the manuscript and companion manuscripts, but involve hospital regions of Paris, Copenhagen and Leipzig during period 2012-2016.
Outcomes	No outcomes were tracked; cross-sectional study.
